# Understanding Photon-Counting CT: Physics, Detector Technology, and Image Reconstructions

**DOI:** 10.3390/s26175574

**Published:** 2026-09-02

**Authors:** Arosh Shavinda Perera Molligoda Arachchige, Fatemeh Darvizeh

**Affiliations:** 1 Postgraduate School of Diagnostic Radiology, University of Milan, Via Festa del Perdono 7, 20122 Milan, Italy; 2 Department of Diagnostic Imaging and Stereotactic Radiosurgery, CDI Centro Diagnostico Italiano S.p.A., Via Simone Saint Bon 20, 20147 Milan, Italy; fatemeh.darvizeh@cdi.it

**Keywords:** photon-counting CT, photon-counting detectors, virtual monoenergetic imaging, iodine quantification, material decomposition, virtual non-contrast imaging

## Abstract

Photon-counting computed tomography (PCCT) represents a detector-level transformation in CT imaging. Unlike conventional energy-integrating detectors, photon-counting detectors directly convert individual X-ray interactions into electrical pulses and classify them according to energy. This architecture enables electronic-noise rejection, smaller detector pixels, improved geometric dose efficiency, and intrinsic spectral acquisition. However, the images available to radiologists are not produced directly by the detector; energy-resolved photon counts must first undergo calibration, correction, projection formation, reconstruction, and material decomposition. This narrative review provides an educational framework linking X-ray attenuation physics, detector materials and architectures, energy thresholds, and detector nonidealities to the resulting PCCT images. It describes conventional polyenergetic and ultra-high-resolution images, virtual monoenergetic imaging, iodine maps, virtual non-contrast imaging, calcium and bone subtraction, virtual non-calcium imaging, effective atomic number maps, electron-density maps, and emerging K-edge techniques. Particular emphasis is placed on the clinical purpose and limitations of each reconstruction, including noise, artifacts, partial-volume effects, misregistration, incomplete subtraction, calibration dependence, and limited cross-platform comparability. Practical considerations for protocol design, image selection, interpretation workflow, and spectral-data archiving are also discussed. Understanding the pathway from photon detection to image formation is essential for selecting the appropriate reconstruction, avoiding misinterpretation, and integrating PCCT effectively into clinical radiology.

## 1. Introduction

Computed tomography (CT) has undergone continuous technical development through multidetector acquisition, faster gantry rotation, wider detector coverage, automated exposure control, iterative reconstruction, dual-energy imaging, and artificial intelligence-based image reconstruction. These advances have improved scan speed, anatomical coverage, dose efficiency, and post-processing capability, but most clinical CT systems still rely on energy-integrating detector (EID) technology [1,2].

In EID-based CT, transmitted X-ray photons are converted into visible light by a scintillator and then into an electrical signal by photodiodes. The detector integrates the total energy deposited during each measurement interval. This architecture produces high-quality anatomical images, but individual photon energies are not preserved, low-amplitude electronic noise contributes to the measured signal, and intrinsic spectral information is limited unless additional acquisition strategies are used.

Photon-counting CT (PCCT) represents a detector-level change in CT technology. Photon-counting detectors use direct-conversion semiconductor materials to convert individual X-ray interactions into electrical pulses. The height of each pulse is related to the deposited photon energy, allowing the detector electronics to count events and assign them to energy ranges using predefined thresholds. This provides the physical basis for electronic-noise rejection, smaller detector pixels, improved geometric dose efficiency, and energy-resolved data acquisition [3].

The imaging consequences of PCCT are therefore not limited to sharper anatomical images. Energy-resolved detection supports spectral and material-specific reconstruction, including virtual monoenergetic imaging, iodine quantification, virtual non-contrast imaging, calcium and bone subtraction, effective atomic number estimation, electron density mapping, and potential K-edge applications. These outputs depend on detector physics, threshold design, spectral calibration, correction algorithms, and reconstruction models. The purpose of this review is to explain how photon-counting detector technology influences CT image formation, spectral and quantitative reconstructions, and their interpretation and limitations. The scope of this review is restricted to medical imaging applications of photon-counting CT; non-medical applications, including non-destructive testing and preclinical or small-animal imaging, are outside its scope.

## 2. Literature Search Strategy

A narrative literature search was performed using PubMed/MEDLINE, Scopus, Web of Science, IEEE Xplore, and Google Scholar, covering publications from database inception through 20th July 2026. Search terms included combinations of “photon-counting CT,” “photon-counting detector,” “spectral CT,” “charge sharing,” “pulse pile-up,” “material decomposition,” “virtual monoenergetic imaging,” “virtual non-contrast,” “iodine quantification,” and “effective atomic number.” Peer-reviewed original studies and technical or clinical review articles were considered when they contributed to the understanding of PCCT detector physics, energy-resolved acquisition, image reconstruction, quantitative imaging, spectral applications, or clinical interpretation. Publications not substantively addressing these topics, non-peer-reviewed material, and articles lacking sufficient technical or clinical relevance to the objectives of the review were not prioritized. Reference lists of relevant articles were also examined to identify additional pertinent publications. Because this was a narrative rather than a systematic review, formal systematic screening, study-selection counts, and a PRISMA-style flow diagram were not undertaken.

## 3. Fundamentals of X-Ray Attenuation and Spectral Imaging

PCCT builds on the same attenuation physics as conventional CT but preserves more information about the energy dependence of that attenuation. In conventional single-energy CT, the transmitted polychromatic spectrum is compressed into a single attenuation value per voxel, displayed as a Hounsfield unit (HU). This value is clinically useful but represents an averaged response to a broad X-ray spectrum and does not uniquely define tissue composition [4]. Spectral CT methods exploit the fact that iodine, calcium, water, fat, soft tissue, uric acid, and other materials attenuate photons differently across the diagnostic energy range.

### 3.1. Conventional CT Acquisition and Reconstruction: Why It Matters for PCCT

A CT image is reconstructed from projection data rather than directly photographed anatomy. During acquisition, the X-ray tube and detector array rotate around the patient and acquire transmission measurements from multiple angular positions. Each measurement records the fraction of the incident beam that remains after passing through a specific path in the body. Reconstruction algorithms estimate the spatial distribution of linear attenuation coefficients that best explain these projection measurements.

**Figure 1:**
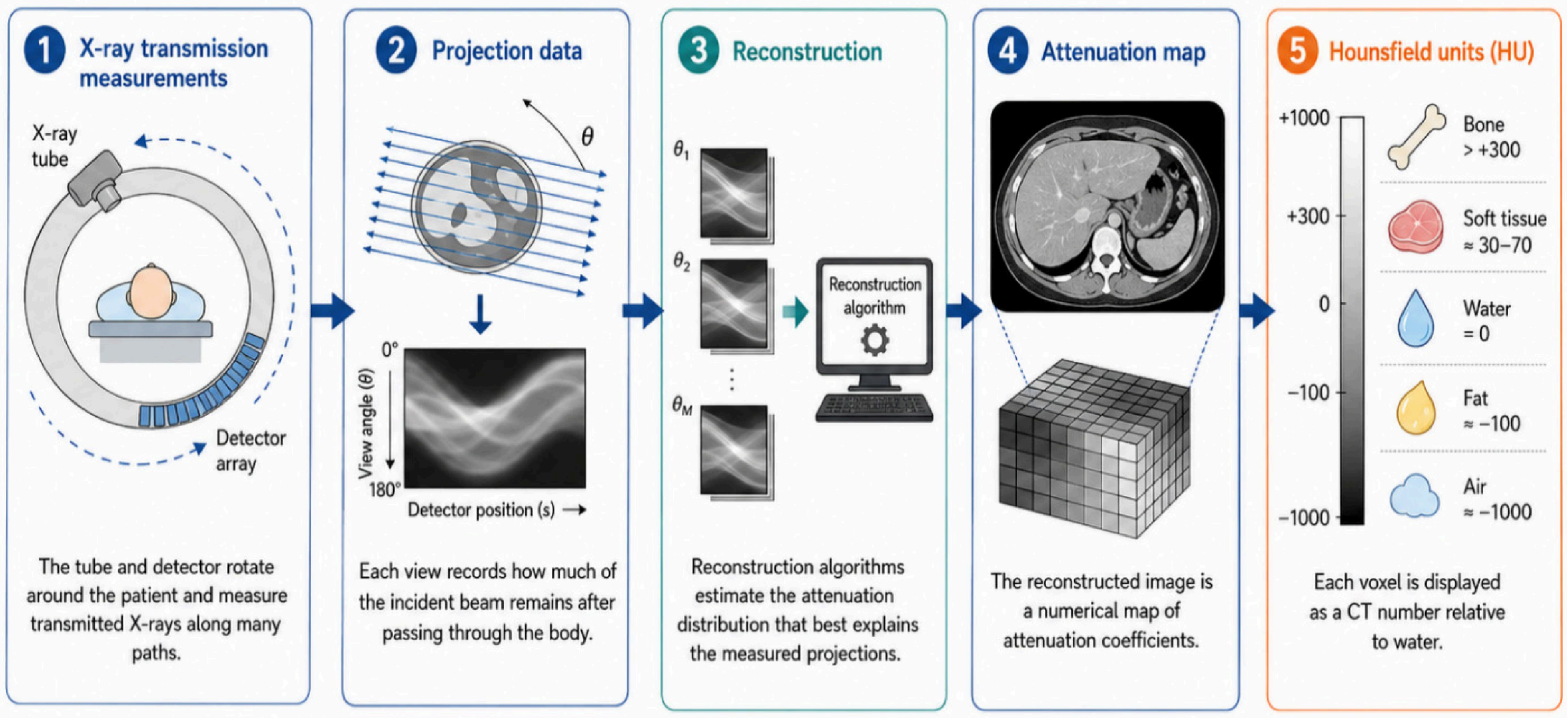
. Formation of a conventional CT image. X-ray transmission measurements acquired from multiple projection angles are reconstructed into a spatial attenuation map, with each voxel displayed as a Hounsfield unit relative to water.

The reconstructed attenuation map is displayed in Hounsfield units, with water assigned 0 HU and air approximately −1000 HU. Materials with greater attenuation than water have positive values. The HU scale is central to clinical CT interpretation, but it is also a simplification because a single HU value reflects the combined effect of photon energy, material composition, beam spectrum, and reconstruction method [5] (Figure 1).

Projection acquisition depends on scanner geometry. In axial scanning, the table is stationary during each rotation and then advances. In the most used technique nowadays, helical scanning, gantry rotation and table movement occur continuously, producing a volumetric dataset [6,7,8]. Pitch describes the relationship between table travel and beam or detector width; higher pitch increases acquisition speed but can increase interpolation demands, whereas lower pitch increases overlap and sampling at the cost of radiation exposure [9].

**Figure 2:**
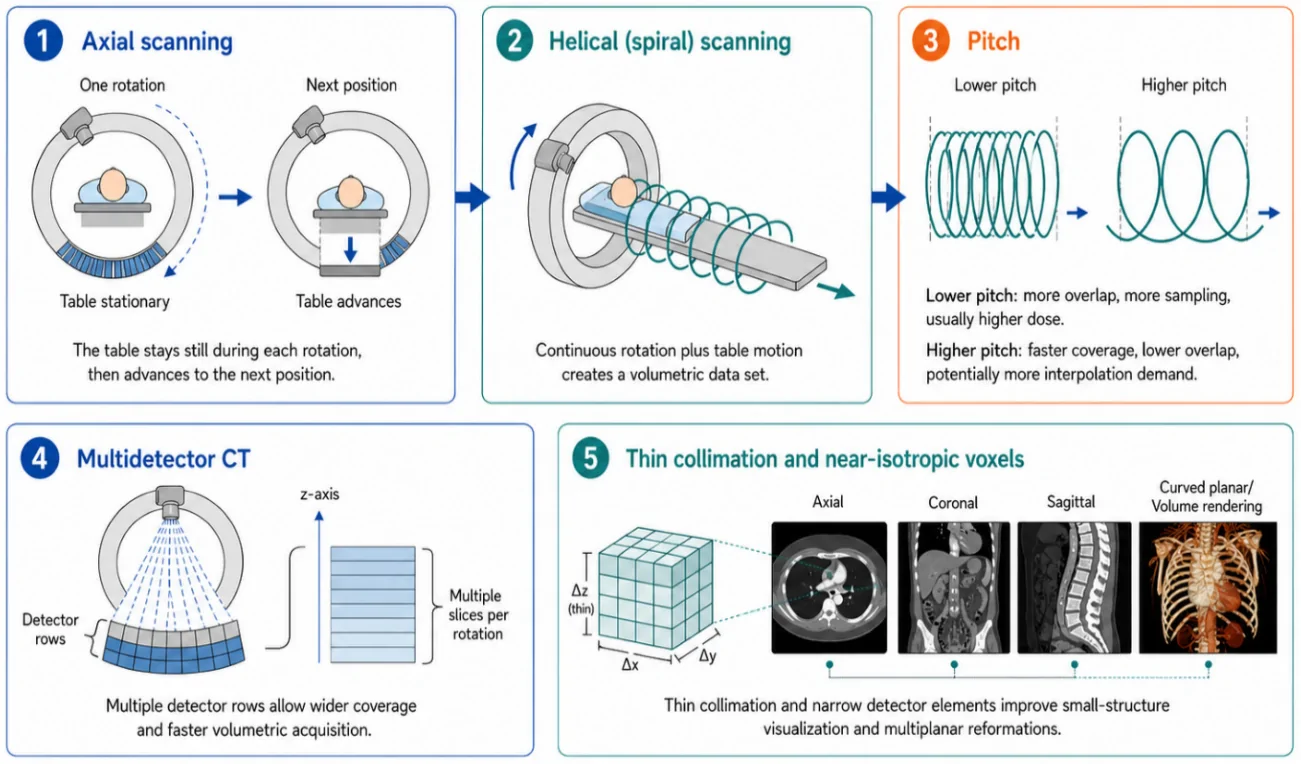
CT acquisition geometry and volumetric sampling. Axial and helical acquisition, pitch, multidetector coverage, and thin collimation determine volumetric sampling, scan speed, and the quality of multiplanar and three-dimensional reconstructions.

Multidetector CT, just as PCCT, uses detector rows along the z-axis to acquire multiple slices per rotation. Thin collimation and narrow detector elements enable near-isotropic voxels, improving multiplanar reformations, curved planar reformations, volume rendering, and small-structure visualization. These conventional principles are directly relevant to PCCT because smaller detector pixels, thinner slices, and ultra-high-resolution reconstruction alter the balance between spatial resolution, noise, and dose efficiency (Figure 2) [10,11,12].

**Figure 3:**
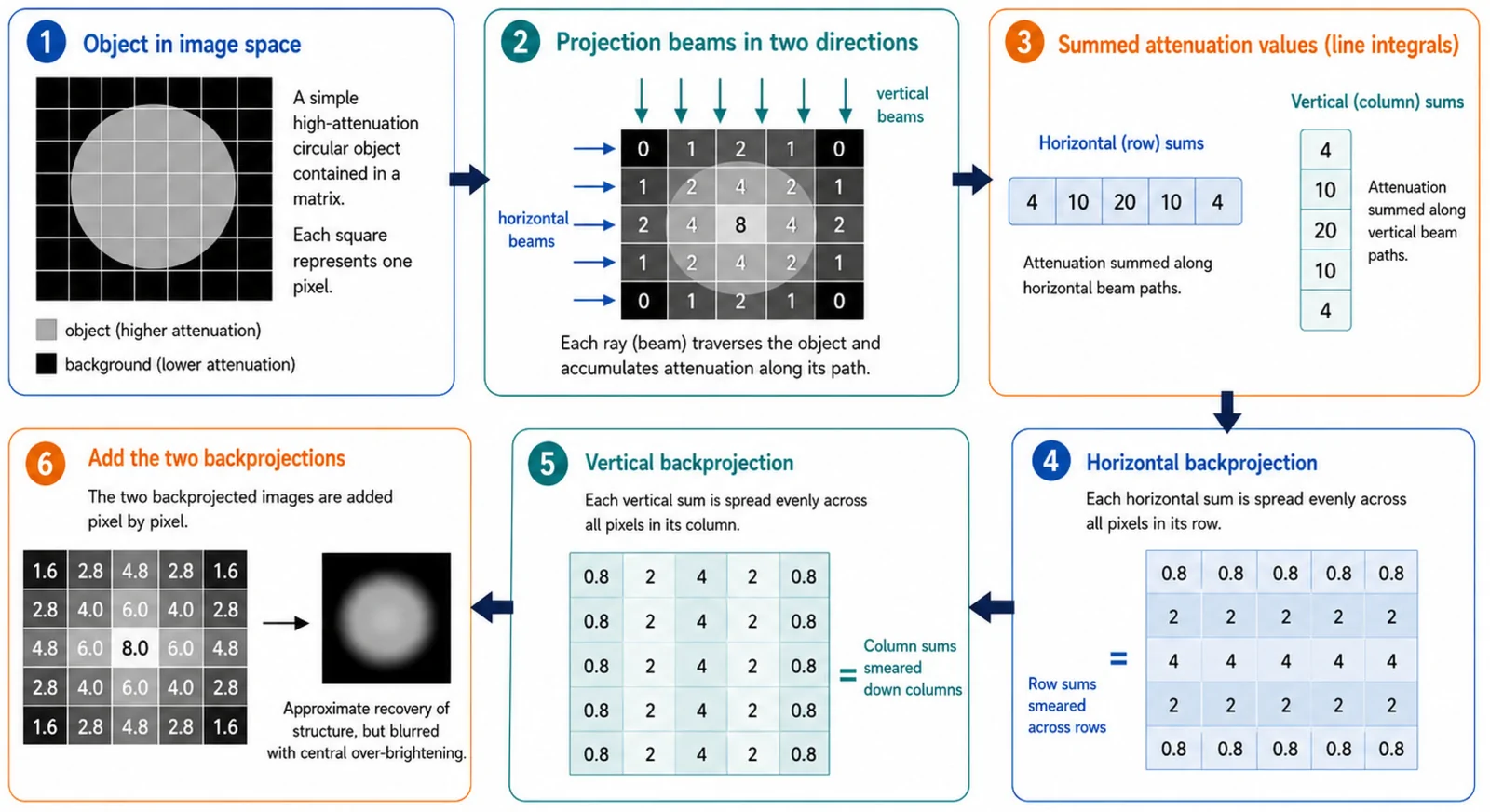
Principle of simple backprojection. Projection measurements are spread back along their acquisition paths and summed to approximate the original object, producing a blurred image that requires filtering for sharper reconstruction.

**Figure 4:**
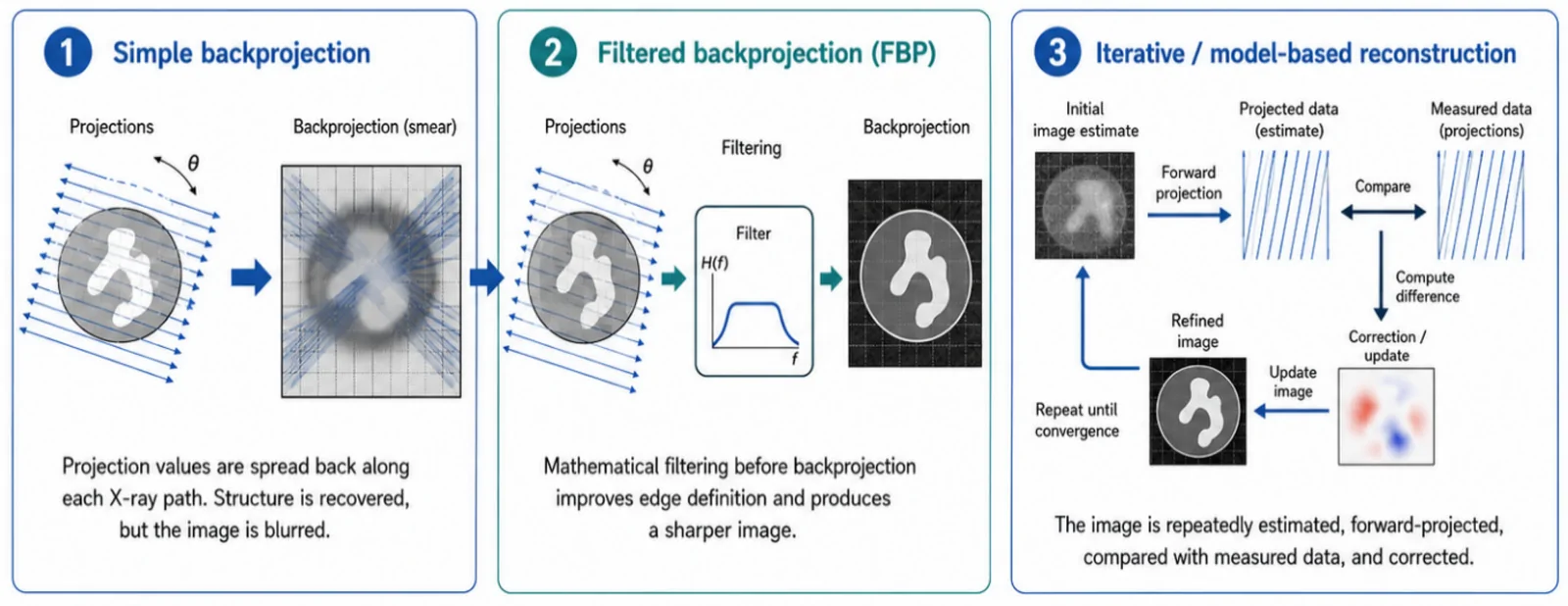
CT reconstruction methods. Simple backprojection produces a blurred image, filtered backprojection improves sharpness by filtering projection data before reconstruction, and iterative or model-based methods repeatedly compare estimated with measured projections to refine the image.

CT reconstruction can be introduced through backprojection. If measured attenuation along each X-ray path is simply distributed back across the image matrix, object location can be approximated, but the image is blurred because each projection contributes signal along an entire path. Filtered backprojection improves sharpness by applying mathematical filtering before backprojection [13]. Iterative and model-based reconstruction methods instead begin with an image estimate, forward-project it, compare the calculated projections with measured data, and update the image repeatedly [14]. In PCCT, this reconstruction process must account not only for attenuation data, but also for photon counts, energy bins, detector response, threshold behavior, and correction algorithms (Figure 3 and Figure 4).

Image quality in CT is governed by spatial resolution, contrast resolution, noise, artifacts, and dose. Spatial resolution is influenced by focal spot size, detector size, sampling, reconstruction kernel, field of view, matrix size, and slice thickness. Noise is affected by photon statistics, tube current, tube voltage, patient size, slice thickness, reconstruction method, and electronic noise. PCCT modifies these image-quality relationships by rejecting low-amplitude electronic noise, permitting smaller detector elements with reduced optical cross-talk, and preserving energy-dependent attenuation information. These features can improve dose efficiency and spatial resolution while enabling spectral reconstructions and material-specific imaging.

Conventional artifacts remain important in PCCT. Beam hardening occurs when low-energy photons are preferentially removed as the beam passes through dense tissue, increasing the mean beam energy and producing cupping or streak artifacts. Photon starvation occurs when too few photons reach the detector, particularly through highly attenuating regions [15,16,17]. Partial-volume effects occur when a voxel contains more than one tissue type, causing the displayed attenuation to represent an average. PCCT can reduce some manifestations of these artifacts, for example through high-keV virtual monoenergetic reconstruction or thinner slices, but it also introduces detector-specific sources of error such as charge sharing, pulse pile-up, K-escape, threshold instability, and count-rate limitations.

**Figure 5:**
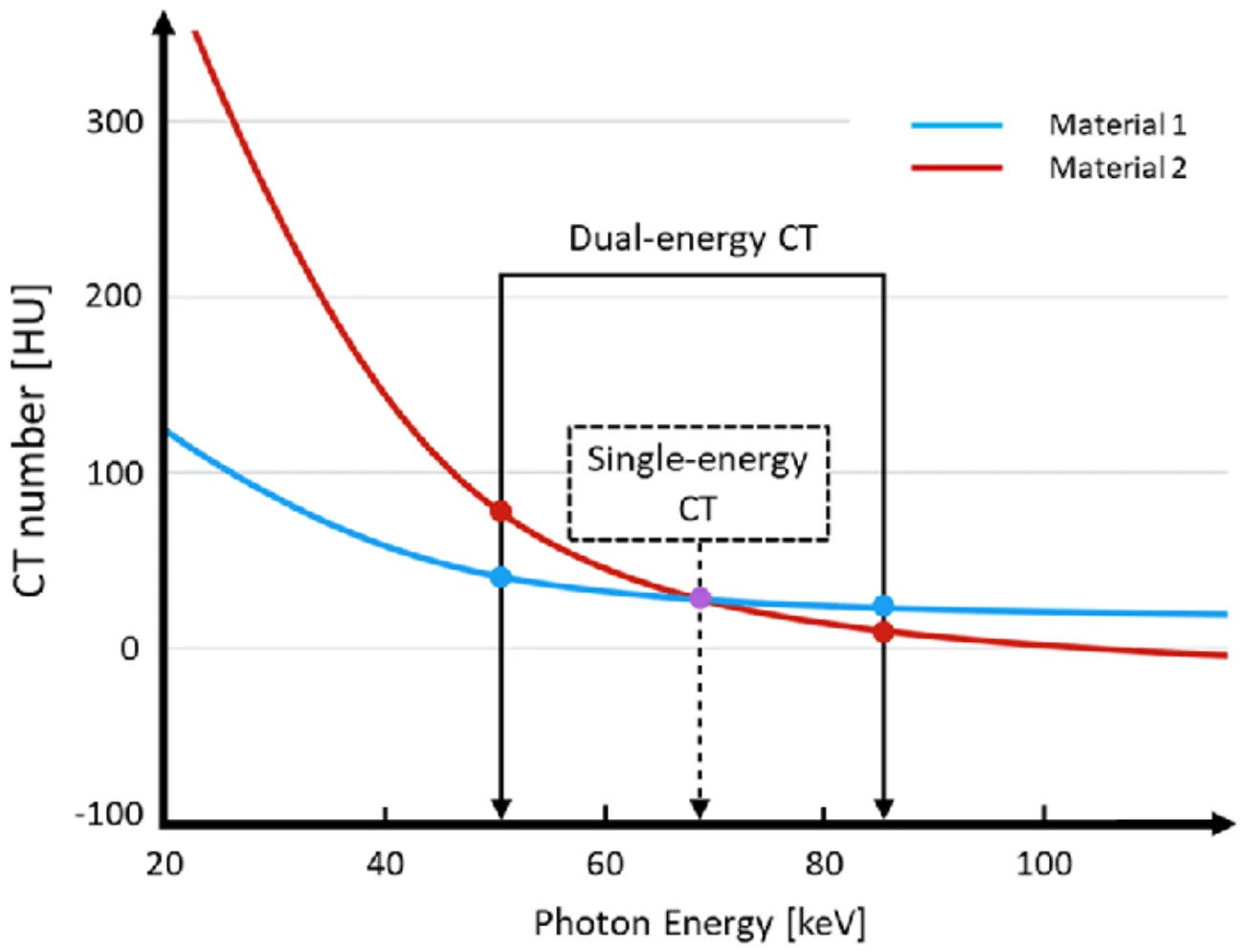
On conventional single-energy CT images, two materials can often not be distinguished due to considerable overlap in their CT numbers. On dual-energy CT scans, materials with different elemental compositions can be differentiated and quantified by comparing their CT numbers at two different energy levels [18].

Dual-energy CT provides the closest conceptual precursor to PCCT spectral imaging. Conventional single-energy CT produces one effective attenuation value per voxel, whereas dual-energy CT samples attenuation using two spectra to support material decomposition and virtual monoenergetic reconstruction (Figure 5). PCCT extends this approach by sorting detected photons into energy ranges at the detector level, allowing energy-resolved data to be obtained from a single acquisition [5].

### 3.2. X-Ray Spectrum in CT

CT uses a polychromatic X-ray beam composed of photons with a range of energies. The selected tube voltage, expressed in kilovoltage peak (kVp), defines the maximum photon energy rather than the energy of every photon [19]. For example, a 120-kVp acquisition contains a broad distribution of photon energies up to approximately 120 keV. The shape of this spectrum is influenced by tube filtration, bowtie filtration, patient size, and scanner design.

As the beam passes through the patient, photons are attenuated by absorption and scattering. Lower-energy photons are generally attenuated more strongly than higher-energy photons, causing the transmitted beam to become progressively harder [20,21]. In energy-integrating detector CT, the detector records a single integrated signal from the transmitted spectrum. In photon-counting CT, detected photons can be counted and assigned to energy ranges, preserving part of the energy-dependent attenuation information that is lost when all photon contributions are summed into one signal.

### 3.3. Photoelectric Absorption

Photoelectric absorption [22] is an interaction in which an incident X-ray photon is completely absorbed by a bound orbital electron. Part of the photon energy is used to overcome the electron binding energy, and the remaining energy is transferred to the ejected electron as kinetic energy. The interaction is favored at lower photon energies and in materials with higher atomic number.

The probability of photoelectric absorption increases strongly with atomic number and decreases as photon energy increases. In simplified form, it varies approximately with Z^3^/E^3^, where Z is atomic number and E is photon energy. High-atomic-number materials such as iodine and calcium therefore attenuate X-rays more strongly than soft tissues, particularly at lower photon energies.

This mechanism explains the high attenuation of iodinated contrast material, cortical bone, calcification, and other dense structures. It also provides one of the physical bases for spectral CT, because materials with different atomic composition show different attenuation behavior across photon energies.

### 3.4. Compton Scattering

Compton scattering is an interaction in which an incident X-ray photon transfers part of its energy to an outer-shell or loosely bound electron and is deflected from its original path with reduced energy [23]. Unlike photoelectric absorption, the photon is not completely absorbed.

**Figure 6:**
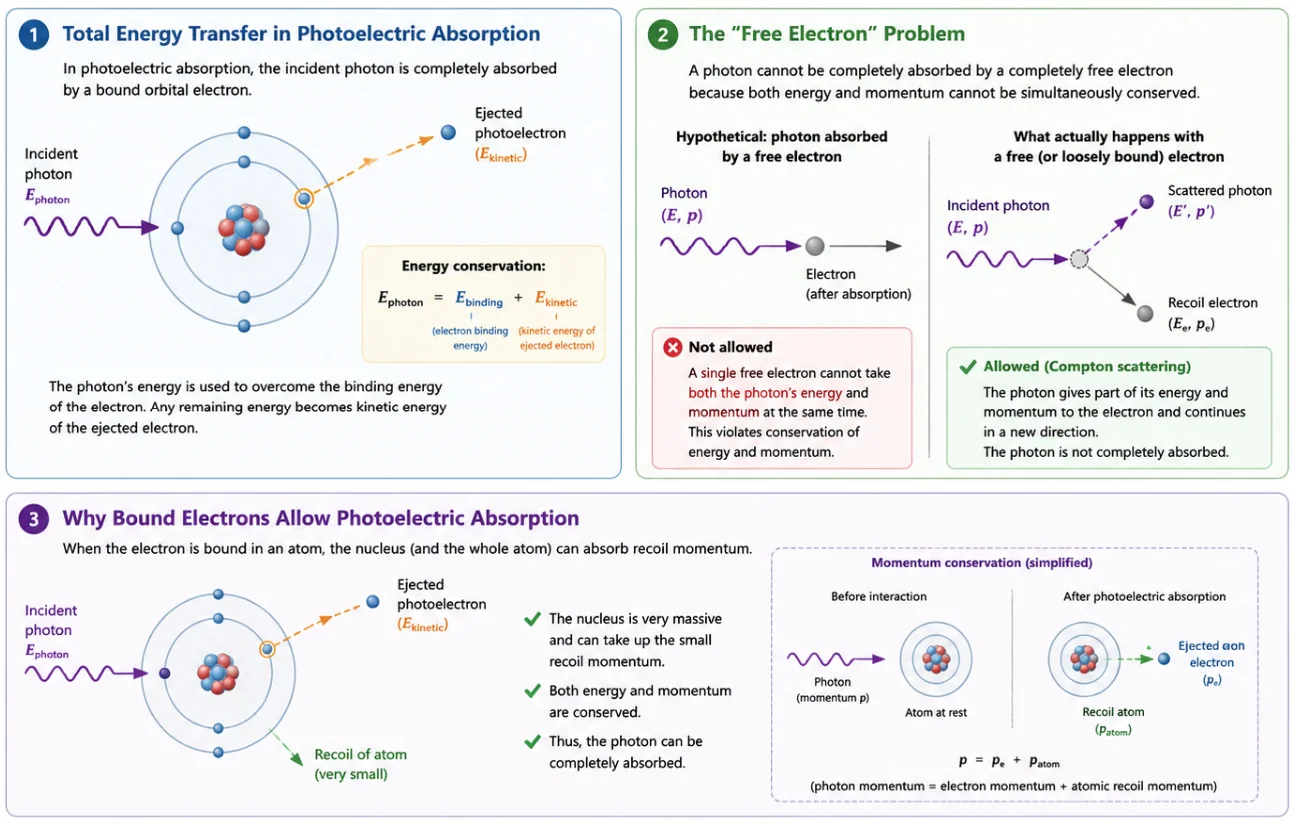
Photoelectric absorption and the role of electron binding. A bound electron can completely absorb an incident photon because the atom absorbs the small recoil momentum, whereas a free electron cannot simultaneously conserve energy and momentum through complete photon absorption.

In the diagnostic CT energy range, Compton scattering is the dominant interaction in most soft tissues. Its probability depends mainly on electron density rather than atomic number. Because many soft tissues have similar electron densities and effective atomic numbers, attenuation differences between soft tissues are often smaller than differences between soft tissue and high-atomic-number materials such as iodine or calcium (Figure 6).

### 3.5. Energy-Dependent Attenuation

The attenuation of a material varies with photon energy. Therefore, a material does not have a single fixed attenuation behavior across the diagnostic spectrum, but rather an energy-dependent attenuation curve. Materials that appear similar on conventional CT may show different attenuation behavior when evaluated at different photon energies [24,25,26].

### 3.6. K-Edge Behavior

K-edge behavior is a specific form of energy-dependent attenuation. Each element has characteristic electron binding energies. When the energy of an incident photon exceeds the binding energy of an inner-shell electron, the probability of photoelectric absorption increases abruptly. This discontinuity is called the K-edge and can act as an element-specific spectral signature [27].

Iodine has a K-edge within the diagnostic energy range and is therefore highly relevant to contrast-enhanced CT. Its K-edge contributes to strong attenuation and supports iodine-specific material decomposition and iodine quantification [28].

## 4. Conventional Energy-Integrating CT Detectors

Most current clinical CT systems use energy-integrating detectors. These detectors are based on an indirect conversion chain: incoming X-ray photons interact with a scintillator, the scintillator converts deposited X-ray energy into visible light, and photodiodes convert this light into an electrical signal. The detector integrates the total deposited energy within each detector element during the measurement interval.

Because EIDs integrate total energy rather than count individual photons, they do not preserve photon-by-photon energy information. A given integrated signal may result from a smaller number of higher-energy photons or a larger number of lower-energy photons. The final measurement therefore represents an averaged response to the transmitted polychromatic spectrum, limiting intrinsic spectral imaging and material decomposition.

Energy weighting is another consequence of EID operation. Since the signal is proportional to deposited energy, higher-energy photons contribute more strongly to the measured signal than lower-energy photons. This weighting is not always ideal because lower-energy photons often carry important contrast information, particularly for iodine-enhanced structures. This contributes to the potential contrast-to-noise advantage of energy-resolving detector systems.

Electronic noise is included in EID measurements because the detector integrates all signal contributions during the measurement interval. This is most important when photon counts are low, such as in low-dose CT, imaging of large patients, or photon-starved regions. Under these conditions, electronic noise can reduce dose efficiency and degrade image quality.

The scintillator-based design also constrains spatial resolution and geometric dose efficiency. Light generated in the scintillator can spread laterally before reaching the photodiode, producing optical cross-talk between neighboring detector elements. Reflective septa reduce this cross-talk but occupy physical detector area, reducing the active absorption area or fill factor [29]. Smaller detector elements can improve spatial resolution, but in scintillator-based detectors they require more septa and are limited by light spread [30].

## 5. Principles of Photon-Counting Detector Operation

**Figure 7:**
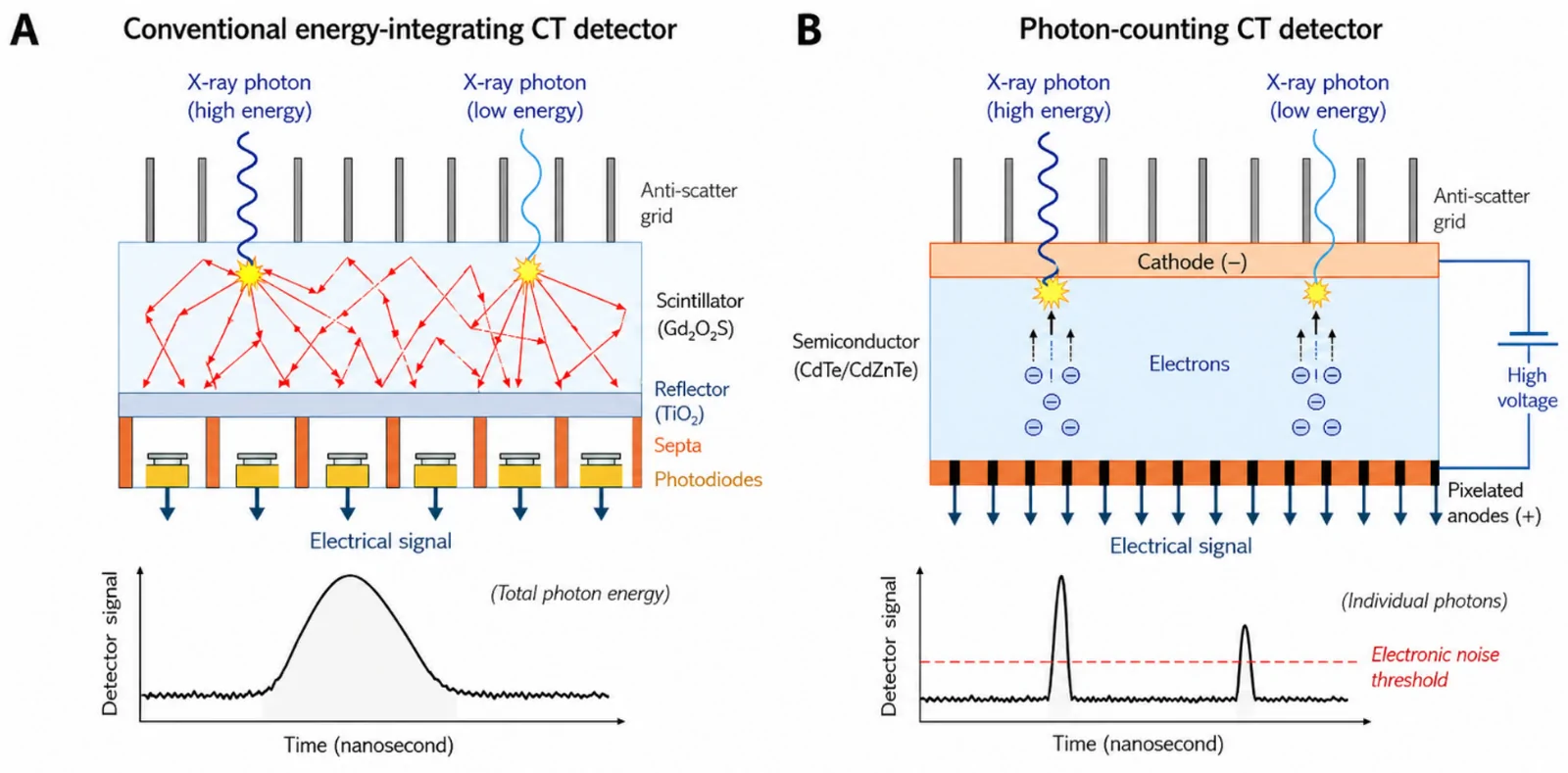
Comparison of energy-integrating and photon-counting CT detectors. (**A**) Energy-integrating detectors convert X-rays to light in a scintillator and integrate the total deposited energy. (**B**) Photon-counting detectors directly convert individual X-ray interactions into electrical pulses, enabling photon counting, energy discrimination, and electronic-noise rejection [3].

PCCT replaces indirect scintillator-photodiode detection with direct-conversion semiconductor detection. Incoming X-ray photons interact within a semiconductor sensor and generate electrical charge without an intermediate light-conversion step. This architecture allows individual photon events to be detected, counted, and assigned to energy ranges when detector conditions are suitable (Figure 7, Table 1).

### 5.1. Direct Conversion

In a photon-counting detector, an absorbed X-ray photon deposits energy in a semiconductor material, producing a cloud of electron–hole pairs. An applied electric field drives electrons and holes toward opposite electrodes, and the movement of these charges induces an electrical pulse in the readout electronics. The absence of a scintillator reduces optical light spread and helps support smaller detector pixels and improved geometric dose efficiency.

### 5.2. Detector Materials

Photon-counting detectors require semiconductor materials that efficiently absorb diagnostic X-ray photons and convert deposited energy into measurable charge. CdTe, CZT, and silicon currently represent the principal detector materials used or actively developed for clinical PCCT, while alternative semiconductors such as GaAs remain under investigation. CdTe and CZT provide high stopping power in relatively compact detector layers because cadmium and tellurium have high atomic numbers. Silicon has lower X-ray absorption efficiency in a conventional face-on geometry but can be used in edge-on designs that increase the photon path length through the sensor.

Detector material influences absorption efficiency, energy resolution, count-rate performance, and spectral accuracy. CdTe and CZT offer compact high-absorption detector modules but are affected by charge sharing, fluorescence escape, polarization, and count-rate limitations. Silicon-based designs may offer favorable charge-transport and count-rate properties but require specific geometries to compensate for lower stopping power [31,32].

Gallium arsenide (GaAs) has also been investigated as a photon-counting detector material and offers greater X-ray absorption efficiency than silicon, with experimental studies demonstrating potential for spectral and multi-energy imaging. However, challenges related to large-scale crystal production, material defects, charge transport, detector uniformity, and thermal stability currently limit its use in clinical PCCT systems [33].

### 5.3. Electron–Hole Pair Generation and Pulse Height

The number of electron–hole pairs generated by a photon interaction is proportional to the energy deposited in the semiconductor. Higher-energy photons generate more charge carriers and therefore produce larger electrical pulses, whereas lower-energy photons generate smaller pulses. Pulse height thus provides an estimate of the photon energy range, although the estimate can be affected by charge sharing, incomplete charge collection, fluorescence escape, and pulse pile-up.

This pulse-height information distinguishes photon-counting detectors from EIDs. EIDs sum deposited energy across many photons, whereas photon-counting detectors attempt to register individual events and classify them by pulse amplitude. This energy-resolving capability is the foundation for material decomposition, iodine quantification, virtual monoenergetic imaging, and effective atomic number estimation [5].

### 5.4. Energy Thresholds and Energy Bins

Energy thresholds are preset pulse-height levels used by the detector electronics. A pulse that exceeds a threshold is counted, whereas a pulse below the lowest threshold can be rejected. Setting the lowest threshold above the electronic-noise level allows low-amplitude electronic noise to be excluded before photon events are counted.

Multiple thresholds create energy bins. A bin is the energy interval between two thresholds or above a specified threshold. For example, photons with pulse heights between two thresholds may be assigned to one energy bin, while photons exceeding a higher threshold may be assigned to another. The number and placement of thresholds vary by detector design, scanner platform, and imaging task. Representative implementations across commercial and experimental PCCT platforms are summarized in Table 2.

Threshold placement affects photon statistics, noise, spectral separation, iodine signal, and material-decomposition accuracy. Lower thresholds increase the number of counted photons but may be more sensitive to unwanted low-energy events or noise. Higher thresholds may improve energy discrimination but reduce photon counts. The resulting energy-bin data provide multiple measurements from the same acquisition, enabling reconstruction algorithms to analyze how attenuation changes across energy ranges [5,55].

The semiconductor sensor operates together with application-specific integrated circuits (ASICs) that provide the pixel-level front-end electronics for charge amplification, pulse shaping, energy-threshold discrimination, counting, and data readout. ASIC design is therefore central to PCCT performance: shorter pulse-processing times improve count-rate capability and reduce pulse pile-up but may increase electronic noise and power consumption, whereas longer shaping times improve noise performance at the expense of greater pulse overlap. Research ASIC families such as Medipix have demonstrated multi-threshold photon counting and charge-sharing correction, although commercial clinical PCCT systems generally employ proprietary detector and readout architectures [56,57,58].

## 6. Detector Materials and Architectures

The performance of PCCT depends on detector material, sensor thickness, pixel size, electrode geometry, septal design, and count-rate capability. These features determine how efficiently photons are absorbed, how accurately photon energy is measured, how much spatial resolution can be achieved, and how reliable spectral measurements are for quantitative reconstruction.

### 6.1. Cadmium Telluride and Cadmium Zinc Telluride Detectors

CdTe and CZT are attractive for clinical PCCT because they have high X-ray absorption efficiency within the diagnostic CT energy range. Their high stopping power allows relatively compact detector layers to absorb a substantial fraction of incident photons, which is important for whole-body CT systems operating at high photon flux.

The same material properties also introduce technical limitations. Charge clouds generated in CdTe or CZT can spread across neighboring pixels, causing charge sharing, double counting, and incorrect energy assignment [42]. Fluorescence photons generated within the detector may escape the original interaction site, producing K-escape or signal misregistration. Polarization effects and count-rate limitations may also influence detector stability and spectral accuracy.

### 6.2. Silicon Detectors

Silicon has lower atomic number and lower stopping power than CdTe or CZT, so a conventional face-on silicon detector would require a greater thickness to absorb diagnostic CT photons efficiently. Edge-on silicon designs address this limitation by allowing X-rays to travel through a longer path within the sensor. This increases absorption efficiency while preserving favorable charge-transport characteristics [42,43].

Potential advantages of silicon-based designs include reduced fluorescence-related effects, good charge transport, and high count-rate performance. Their practical use depends on detector geometry, manufacturing complexity, absorption efficiency, and integration into clinical CT scanner designs.

### 6.3. Pixel Size

Detector pixel size influences spatial resolution, dose efficiency, and spectral accuracy. Smaller detector pixels improve geometric sampling and can increase spatial resolution, particularly for ultra-high-resolution applications. However, small pixels may increase the probability that charge from a single photon interaction spreads into adjacent pixels, producing charge sharing and energy misclassification.

The optimal pixel size is therefore a balance between spatial resolution, photon-counting accuracy, electronic readout performance, charge-sharing correction, and clinical dose efficiency. PCCT can use smaller detector pixels than conventional scintillator-based systems because direct conversion reduces light spread and may require less septal separation [59].

The resulting improvement in spatial resolution can be quantified using the modulation transfer function (MTF): in clinical high-resolution imaging, conventional EID-CT provides approximately 9 lp/cm at the 10% MTF level, whereas PCCT ultra-high-resolution acquisitions have demonstrated values of approximately 16–17 lp/cm, depending on the reconstruction kernel, corresponding to resolvable structures on the order of 0.3 mm [39,40,41].

### 6.4. Detector Septa

In scintillator-based EIDs, septa are used to confine visible light and reduce optical cross-talk between detector elements. These septa occupy detector area that is not active for X-ray absorption, reducing geometric dose efficiency. Photon-counting detectors do not require the same scintillator-light confinement and may reduce septal losses, although detector architectures still require electrical isolation, electrode structures, and module boundaries that influence fill factor [60].

### 6.5. Count-Rate Capability

Clinical CT exposes detectors to very high photon flux, particularly at high tube current or in less attenuating body regions. Photon-counting detectors must process individual pulses rapidly enough to avoid event overlap and counting losses. If photons arrive too close together in time, pulses may overlap, resulting in pile-up, incorrect energy assignment, or nonlinear detector response [61]. Count-rate capability is therefore central to PCCT image quality and quantitative reliability.

### 6.6. Representative Commercial and Experimental PCCT Systems

Photon-counting CT systems differ substantially in detector material, detector geometry, number of energy bins, and threshold implementation. These differences influence count-rate capability, spectral resolution, spatial sampling, and the types of material-decomposition strategies that can be implemented. Table 2 summarizes representative commercial and experimental whole-body PCCT platforms. Importantly, the number and configuration of energy thresholds are platform- and acquisition-mode-dependent, and research configurations should not be assumed to represent routine clinical operating modes.

## 7. Technical Challenges in Photon-Counting CT

PCCT provides energy-resolved photon detection, but its performance is limited by detector nonidealities. These effects influence spatial resolution, spectral accuracy, quantitative reconstruction, and artifact behavior. The most important limitations include charge sharing, pulse pile-up, K-escape, count-rate effects, threshold instability, and residual electronic-noise considerations (Table 3).

### 7.1. Charge Sharing

Charge sharing occurs when the charge cloud generated by a single photon interaction spreads across more than one detector pixel [62]. The event may then be counted in multiple pixels or assigned an energy lower than the true photon energy in each affected pixel. This can reduce spatial and spectral accuracy, particularly in small-pixel detector designs. Correction algorithms can mitigate charge sharing, but residual effects may influence material decomposition and quantitative maps.

### 7.2. Pulse Pile-Up

Pulse pile-up occurs when two or more photons arrive at a detector pixel within a time interval too short for the electronics to separate their pulses. The detector may count them as a single event with incorrectly high energy, may miss one event, or may produce nonlinear count losses. Pile-up is most important at high photon flux and can bias energy-bin data, reduce spectral accuracy, and affect CT number stability [61,63].

### 7.3. K-Escape

K-escape occurs when an incident X-ray photon interacts within the detector material and generates characteristic fluorescence radiation. If the fluorescence photon escapes the original interaction site, the recorded energy at that site is lower than the incident photon energy. If the fluorescence photon is absorbed in a neighboring pixel, it can also produce spatial or spectral misregistration. K-escape is particularly relevant for high-Z detector materials such as CdTe and CZT [34,46,47].

### 7.4. Count-Rate Limitations

Count-rate limitations describe the finite ability of detector electronics to process individual photon events at high flux. When the event rate exceeds detector capability, count losses, pulse overlap, and energy misclassification may occur. These effects can introduce nonlinear detector response and reduce the reliability of quantitative spectral reconstructions. Scanner design, detector material, pixel size, shaping time, and correction algorithms all influence count-rate performance [64].

### 7.5. Threshold Instability and Calibration

Energy thresholds must remain stable and accurately calibrated across detector pixels and over time. If thresholds drift or vary between pixels, photons may be assigned to the wrong energy bin. This can create spatial nonuniformity, affect iodine quantification, alter virtual monoenergetic images, and degrade material-decomposition accuracy. Calibration procedures and correction algorithms are therefore essential components of PCCT system performance.

Long-term detector stability is also influenced by semiconductor polarization and temperature. In CdTe- and CZT-based detectors, charge trapping during sustained irradiation can alter the internal electric field and charge-collection efficiency, while temperature-dependent changes in detector gain and threshold response may affect energy-bin assignment. Stable thermal control and regular automated detector calibration are therefore important for maintaining uniformity and quantitative spectral accuracy, particularly under high photon-flux conditions [65].

### 7.6. Electronic Noise

Photon-counting detectors can reject much low-amplitude electronic noise by setting the lowest threshold above the electronic-noise floor. This is a major advantage over EIDs, which integrate electronic noise into the measured signal. However, electronic noise is not irrelevant in PCCT. Threshold placement, readout electronics, detector stability, and low-signal conditions can still influence counting accuracy, especially when thresholds are set close to the noise floor or when photon statistics are limited [34,46,47].

## 8. From Photon Counts to Projection Data

The distinctive feature of photon-counting CT (PCCT) is that the detector does not simply integrate all deposited X-ray energy into one signal. Instead, the detector counts individual photon events and classifies them according to pulse height, which is related to photon energy. These photon events are sorted into predefined energy bins. Each energy bin therefore contains information about how many photons were detected within a specific energy range during the CT acquisition [49,50,51].

However, raw photon counts cannot be directly interpreted as CT images. Before image reconstruction, the detector data must be corrected and converted into projection data. These corrections are necessary because photon-counting detectors are affected by detector nonidealities such as charge sharing, pulse pile-up, K-escape, count-rate effects, threshold variation, and pixel-to-pixel response differences. Correction algorithms and calibration procedures attempt to compensate for these effects so that the recorded energy-bin counts more accurately represent the transmitted X-ray spectrum [49,50,52].

**Figure 8:**
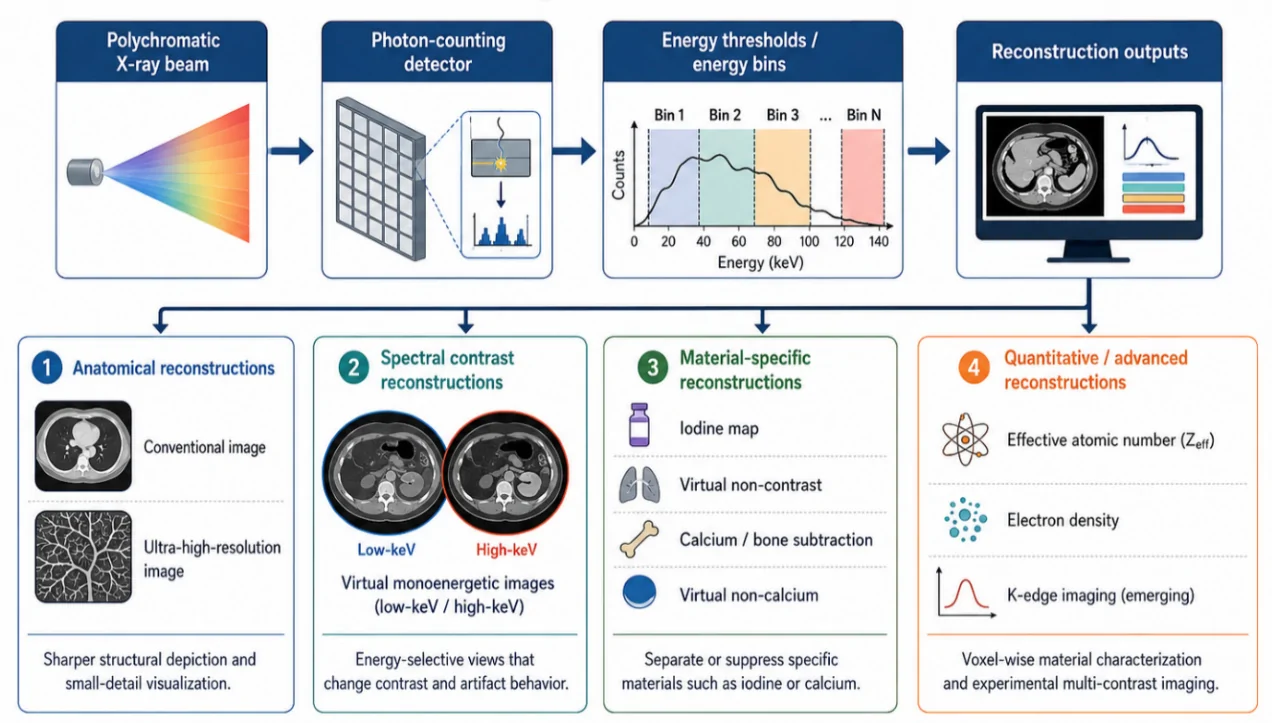
Major reconstruction outputs of photon-counting CT. Energy-resolved photon counts can be reconstructed into conventional and ultra-high-resolution anatomical images, virtual monoenergetic images, material-specific maps, and quantitative outputs such as effective atomic number, electron density, and emerging K-edge images.

After correction, the photon-count data are transformed into projection data. Projection data describe how much the X-ray beam has been attenuated along each path through the patient. In conventional CT, this process produces a single set of projection data that is reconstructed into an anatomical image. In PCCT, projection information may be available separately for different energy bins. This enables several reconstruction strategies (Figure 8): the bin data may be combined to generate conventional CT-like images, reconstructed separately to produce energy-bin images, or processed through material decomposition algorithms to generate material-specific images [49,51,53].

Material decomposition can be performed in projection space or image space. In projection-domain decomposition, material separation is performed before image reconstruction, using the energy-dependent attenuation information contained in the projection data. This approach can be physically accurate because it models attenuation before reconstruction, but it is technically more complex and requires careful system calibration. In image-domain decomposition, images are reconstructed first, and material separation is then performed using reconstructed images from different energy bins or virtual energy levels. This approach is easier to implement but may be more sensitive to beam-hardening effects, image noise, and reconstruction-related biases [53].

The important concept is that the detector does not directly “make” iodine maps, virtual monoenergetic images, or virtual non-contrast images. The detector first records energy-resolved photon counts. These counts are corrected, converted into projection data, and then reconstructed or decomposed into different image types. The final PCCT images seen at the workstation are therefore the result of a chain that begins with photon detection and ends with reconstruction algorithms.

This chain explains why PCCT reconstructions should be interpreted as complementary outputs rather than interchangeable images. A conventional anatomical image, a low-keV virtual monoenergetic image, an iodine map, and a virtual non-contrast image may all be derived from the same acquisition, but each emphasizes different physical information and each has specific pitfalls. Understanding this relationship is essential for using PCCT appropriately in clinical practice.

### 8.1. Anatomical Reconstructions

#### 8.1.1. Conventional Polyenergetic Images

Conventional polyenergetic images are CT-like reconstructions generated from photon-counting CT (PCCT) data to provide a familiar anatomical image appearance. Although the acquisition is performed using photon-counting detectors, energy-resolved data from multiple bins are combined, often with scanner- and task-specific weighting, to produce images that resemble routine single-energy CT images acquired at a selected tube voltage [49,51,66].

These images usually form the baseline series for clinical interpretation. They display soft tissue, fat, air, bone, blood, contrast enhancement, calcification, and metal in a format that is familiar from conventional CT, allowing routine anatomical assessment and comparison with prior examinations. At the same time, they may benefit from detector-level advantages of PCCT, including reduced electronic noise, improved geometric dose efficiency, and higher spatial resolution, depending on the scanner design, reconstruction mode, kernel, slice thickness, and dose level [49,50,66].

However, conventional polyenergetic images do not fully display the spectral information contained in the PCCT acquisition. Because the energy-bin data are combined into a single anatomical image, material-specific information may be partially obscured. Subtle iodine enhancement, iodine-calcium separation, virtual non-contrast information, effective atomic number differences, or perfusion-related iodine defects may be more apparent on dedicated spectral reconstructions than on the conventional image alone.

For this reason, conventional polyenergetic images should be considered the anatomical reference rather than the endpoint of PCCT interpretation. In pulmonary vascular imaging, iodine maps may reveal perfusion abnormalities; in abdominal imaging, low-keV virtual monoenergetic images may improve lesion conspicuity; and in vascular or musculoskeletal imaging, high-keV or calcium-subtracted images may reduce artifacts or clarify calcified structures. Conversely, spectral reconstructions should not be interpreted in isolation, because material maps and virtual reconstructions may be affected by noise, subtraction errors, motion, misregistration, or reconstruction-specific bias. A practical workflow is therefore to begin with the conventional polyenergetic series for anatomical assessment and then use spectral reconstructions selectively to answer specific diagnostic questions.

#### 8.1.2. Ultra-High-Resolution Reconstructions

**Figure 9:**
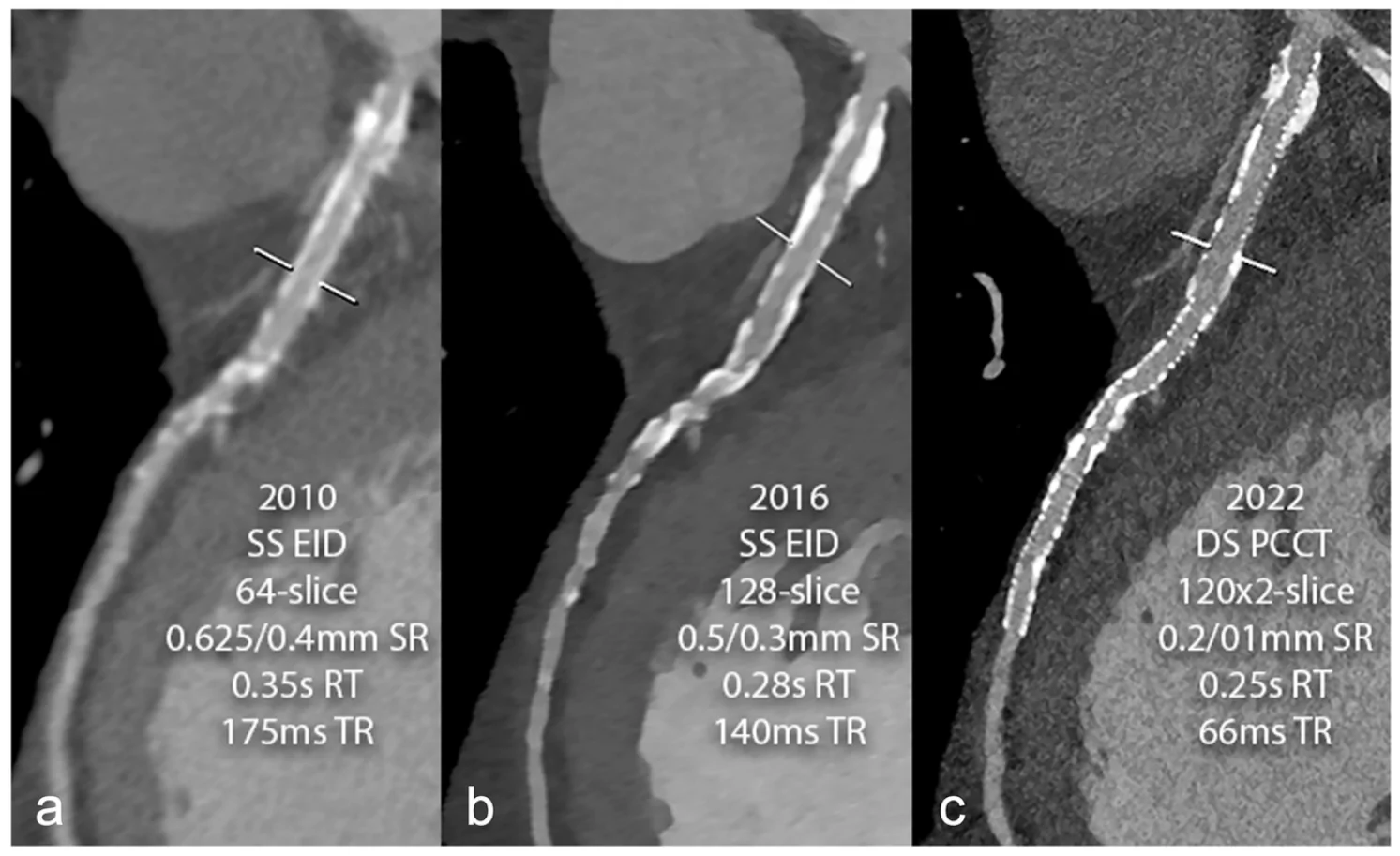
Clinical evolution of CCTA for coronary stent assessment over 15 years in the same approximately 70-year-old patient with coronary artery disease and multiple LAD stents. (**a**) 2010 single-source 64-slice CT (0.625-mm slices; 175-ms temporal resolution). (**b**) 2016 single-source 128-slice CT (0.5 mm; 140 ms). (**c**) 2022 dual-source photon-counting CT (0.2 mm; 66 ms). Progressive improvements in image sharpness and reductions in motion and blooming artifacts from stents and calcifications are evident. CCTA, cardiac CT angiography; LAD, left anterior descending artery; PCCT, photon-counting CT; SS, single source; DS, dual source [59].

Ultra-high-resolution (UHR) reconstructions exploit the smaller detector pixels and reduced light spread of PCCT, together with thin collimation, thin sections, and sharp reconstruction kernels, to improve visualization of fine anatomical detail. On the currently available dual-source NAEOTOM Alpha platform, the Quantum HD mode permits reconstruction at slice thicknesses as low as 0.2 mm, with an in-plane spatial resolution of approximately 0.11 mm. The system incorporates a 0.4 × 0.5 mm microfocal spot to support the high spatial resolution provided by the photon-counting detector. These specifications are platform-specific and should not be interpreted as universal characteristics of all photon-counting CT systems. These high-resolution capabilities are particularly useful when diagnosis depends on small structures, including lung interstitium and bronchioles, temporal bone anatomy, cortical and trabecular bone, coronary arteries and stents, small vessels, urinary stones, and postoperative or implant-related anatomy [37,38]. In these settings, UHR imaging may improve depiction of subtle reticulation, small nodules, fracture lines, erosions, calcifications, trabecular architecture, stone morphology, and stent or luminal detail (Figure 9).

The main trade-off is increased image noise, particularly with very thin sections and high-spatial-frequency kernels. This may produce a grainier appearance and reduce low-contrast soft-tissue conspicuity, especially in low-dose examinations and larger patients. Smaller detector pixels may also increase charge-sharing effects if correction is inadequate. UHR reconstructions should therefore be used as targeted supplementary series rather than replacements for standard soft-tissue images, with section thickness, kernel, dose, and reconstruction settings tailored to the clinical question. Their selective use also limits unnecessary reconstruction burden, storage requirements, and interpretation time [67,68].

### 8.2. Spectral Contrast Reconstructions

#### Virtual Monoenergetic Imaging

Virtual monoenergetic imaging (VMI) is one of the most clinically important spectral reconstruction techniques in PCCT. Conventional CT images are generated from a polychromatic X-ray spectrum, meaning that the final image represents the combined attenuation effect of photons with many different energies. In contrast, VMI reconstructs images as if they had been acquired using photons of a selected single energy level, expressed in kiloelectron volts (keV). These images are not acquired as separate scans; they are mathematically reconstructed from the energy-resolved data obtained during the same acquisition [49,51,53].

VMI is generated from energy-bin data by using reconstruction algorithms that model the energy-dependent attenuation behavior of tissues and materials. These algorithms estimate how the scanned anatomy would appear at a selected monochromatic energy, commonly using material decomposition based on basis materials such as iodine and water or on photoelectric and Compton components [50,53]. The selected keV level determines the image appearance. Low-keV reconstructions increase attenuation of materials with strong low-energy absorption, especially iodine, whereas high-keV reconstructions reduce the effect of highly attenuating materials and may decrease beam-hardening or metal-related artifacts.

Low-keV VMI increases iodine conspicuity and can improve contrast-to-noise ratio for iodine-enhanced anatomy and pathology. This is useful in CT angiography, including pulmonary embolism imaging, coronary CTA, aortic CTA, carotid CTA, peripheral CTA, and other vascular protocols [69,70,71]. Low-keV reconstructions may also help in patients with suboptimal contrast bolus timing or reduced iodine dose, because they can increase vessel-to-background contrast. In abdominal imaging, low-keV VMI may improve conspicuity of enhancing lesions, hypervascular tumors, pancreatic lesions, renal lesion enhancement, bowel wall enhancement, and ischemic bowel [4,72,73,74]. It may also support reduced-contrast protocols, although the extent of iodine dose reduction depends on scanner type, patient habitus, protocol, body region, and diagnostic indication [75,76,77].

The main limitation of low-keV VMI is noise. At very low energy levels, iodine contrast increases, but image noise and artifacts may also become more conspicuous, especially in large patients or low-dose examinations. Low-keV images can also exaggerate the visual appearance of enhancement, so conventional single-energy HU thresholds should not be applied directly unless validated for the selected keV level. Beam-hardening, photon-starvation, and reconstruction artifacts may also be more apparent at very low keV, making comparison with conventional images, iodine maps, and other spectral reconstructions important.

Advanced iterative and deep-learning-based reconstruction techniques may partially mitigate the increased noise encountered at low virtual monoenergetic energy levels. Importantly, neural-network processing can occur at different stages of the spectral reconstruction chain rather than necessarily operating directly on raw detector counts. Depending on the implementation, algorithms may process energy-resolved projection data, reconstructed energy-bin or material-basis images, or the final virtual monoenergetic images. By learning spatial and spectral correlations across these inputs, neural networks can suppress stochastic noise while attempting to preserve edges and material-dependent attenuation information. In photon-counting CT, experimental approaches have used deep learning to approximate computationally intensive iterative reconstruction and to denoise high-resolution spectral images using lower-noise reconstructions as prior information [78,79,80]. These strategies are particularly relevant to low-keV VMI, where increased iodine attenuation improves vascular and lesion contrast but is accompanied by greater image noise. However, the degree of noise reduction and preservation of quantitative spectral information depends on the network architecture, training data, reconstruction domain, and scanner implementation, and aggressive denoising may alter image texture or quantitative measurements.

High-keV VMI is primarily useful for artifact reduction. Higher-energy virtual images are less affected by strong attenuation from dense materials such as metal, bone, dense contrast, and calcification. Applications include imaging near hip prostheses, spinal hardware, shoulder arthroplasty, dental materials, trauma implants, and oncologic hardware. High-keV reconstructions may also help in regions prone to beam-hardening artifacts, including the posterior fossa, skull base, shoulders, pelvis, and areas with dense intravenous contrast [74,81]. In chest CTA, for example, high-keV images may reduce artifacts from dense contrast in the superior vena cava [82].

**Figure 10:**
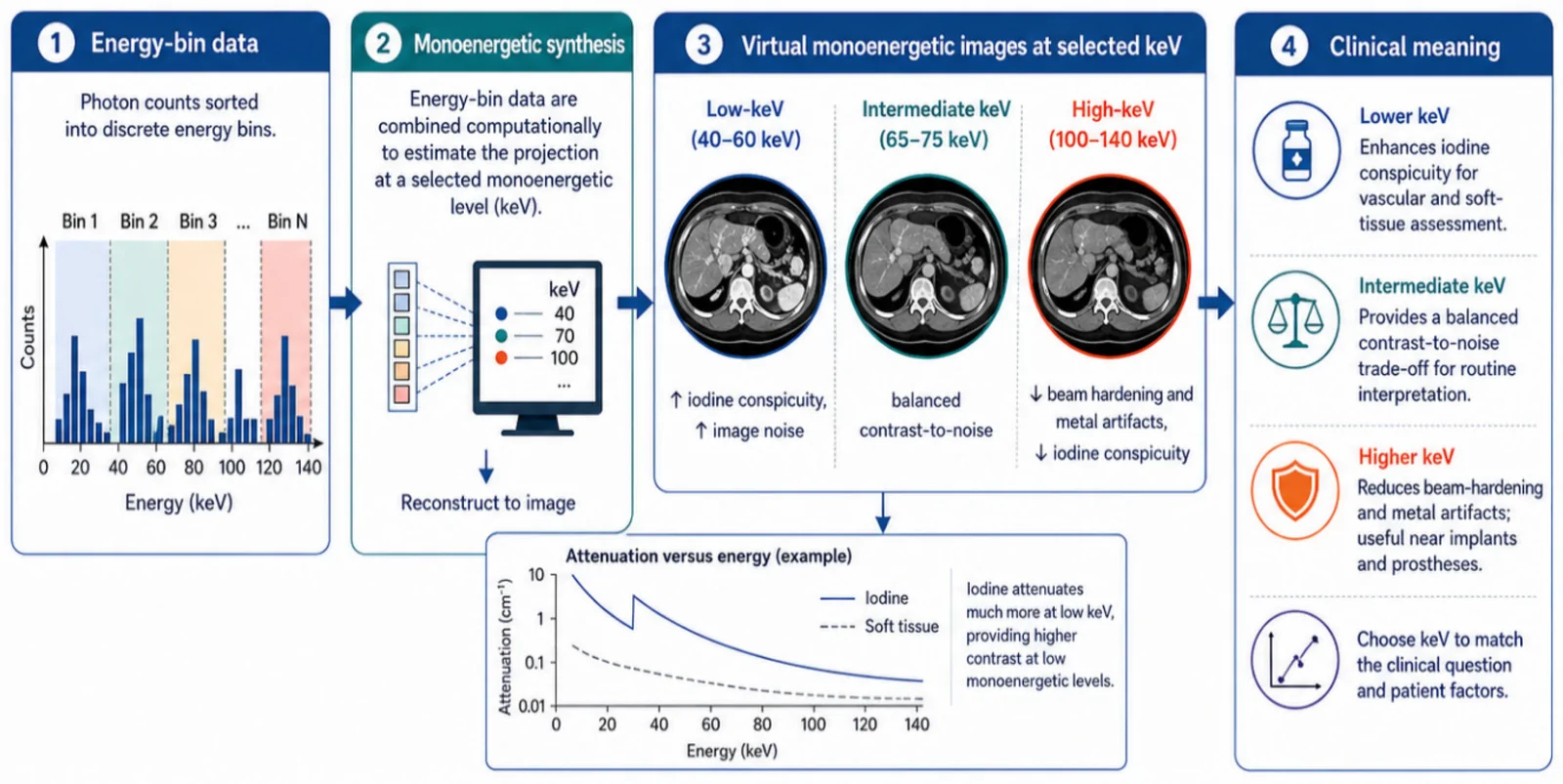
Virtual monoenergetic imaging in photon-counting CT. Energy-bin data are computationally combined to synthesize images at selected keV levels: low-keV images increase iodine conspicuity but may increase noise, intermediate-keV images provide balanced routine contrast, and high-keV images reduce beam-hardening and metal-related artifacts.

The major limitation of high-keV VMI is reduced iodine conspicuity. Vessels, enhancing lesions, inflammatory changes, and subtle perfusion abnormalities may become less visible because iodine attenuation decreases at higher photon energies. Therefore, high-keV images are best used as artifact-reduction reconstructions rather than as the primary series for detecting subtle enhancement. In practice, the optimal VMI energy depends on the anatomical region, patient size, contrast phase, diagnostic task, and desired balance between contrast, noise, and artifact reduction (Figure 10).

### 8.3. Material Density Images and Multi-Material Decomposition

Material density images estimate the density or contribution of selected basis materials within the scanned volume. Examples include iodine density images, calcium density images, water density images, fat density images, uric acid images, hydroxyapatite images, and soft-tissue basis images. Instead of representing each voxel only by a single attenuation value, material decomposition estimates how much of the voxel behaves like one or more selected materials [63,83,84].

These images are generated by spectral material decomposition. Because different materials have different energy-dependent attenuation curves, reconstruction algorithms can estimate the contribution of selected basis materials. Conventional dual-energy CT commonly performs two-material decomposition. PCCT, by acquiring multiple energy bins, may support more advanced multi-material decomposition, although this remains technically challenging. At present, such advanced multi-material decomposition should be regarded primarily as an emerging or investigational capability rather than a standardized routine clinical application.

**Figure 11:**
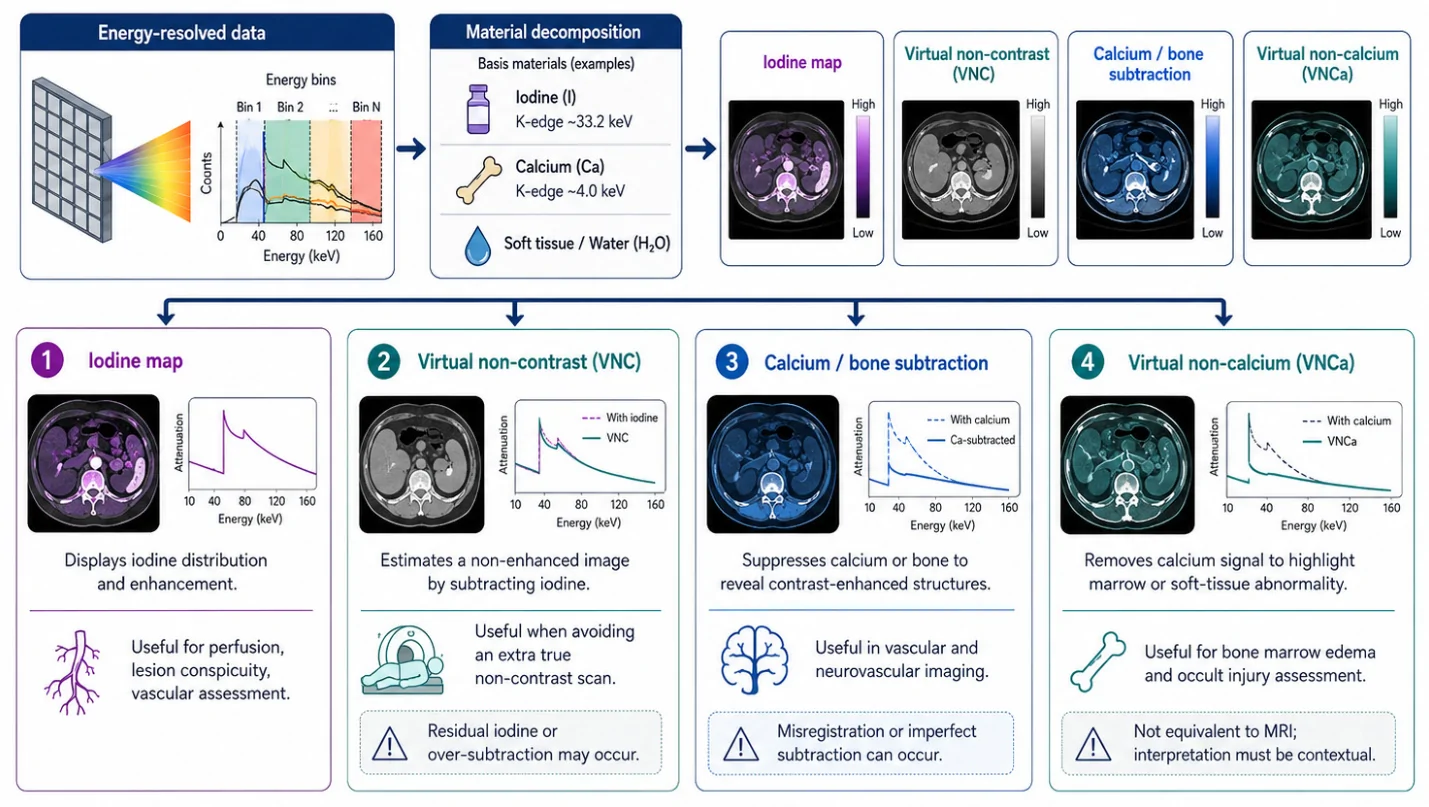
Material-specific reconstructions in photon-counting CT. Energy-resolved data are decomposed into basis materials to generate iodine maps, virtual non-contrast images, calcium- or bone-subtracted images, and virtual non-calcium images, each providing task-specific information while remaining susceptible to subtraction and misregistration artifacts.

The selected material basis is central to interpretation. An iodine–water model is optimized for iodine enhancement; a calcium–iodine model may be more useful in vascular calcification; a uric acid–calcium model may support stone characterization; and a hydroxyapatite model may support bone mineral assessment. Material decomposition is therefore task-specific rather than universally applicable (Figure 11).

Clinical applications include renal stone composition, gout, bone mineral assessment, vascular plaque characterization, tumor enhancement, liver fat and iodine assessment, contrast quantification, and differentiation of iodine from calcium or hemorrhage. In urolithiasis, material decomposition may help distinguish uric acid from non-uric-acid stones. In gout, it may support identification of urate deposition. In vascular imaging, calcium and iodine decomposition may help separate calcified plaque from contrast-enhanced lumen. In oncology, iodine density images may support tumor enhancement quantification and treatment response assessment [30,85,86,87,88].

The main limitation is dependence on the selected material model. If the wrong basis materials are chosen, the output may be misleading. Multi-material decomposition is also more complex than two-material decomposition because more material components must be separated from noisy spectral data. Mixed voxels, beam hardening, motion, detector nonidealities, and partial volume can all affect accuracy. Quantitative values should not be assumed to be interchangeable across vendors, protocols, thresholds, or reconstruction algorithms.

#### 8.3.1. High-keV VMI and Metal Artifact Reduction Algorithms

**Figure 12:**
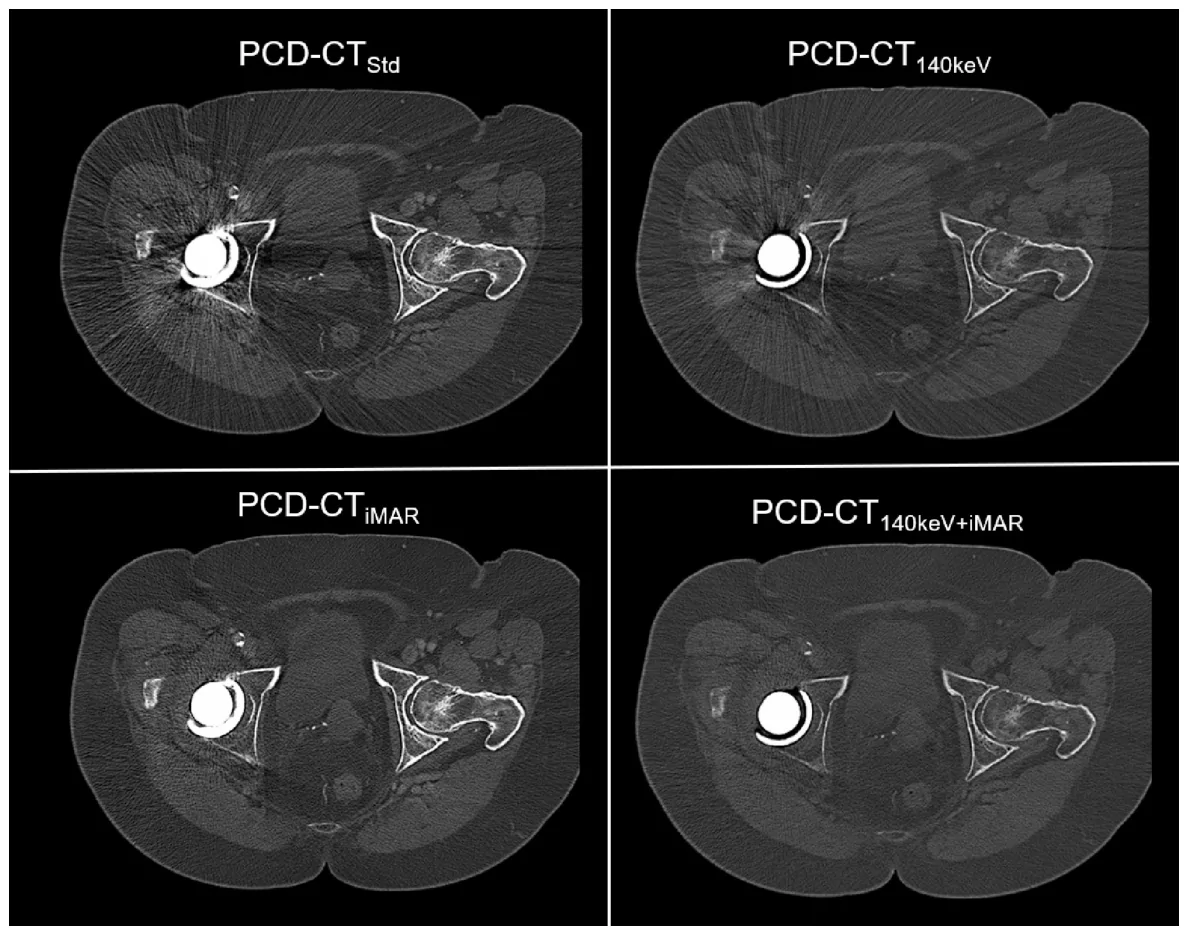
Example images from a 71-year-old patient with non-small cell lung cancer and a right hip prosthesis. PCD-CT at 140 keV achieved only limited metal artifact reduction, whereas iMAR alone and iMAR combined with 140-keV virtual monoenergetic reconstruction produced substantially greater artifact suppression. PCD-CTStd indicates standard reconstruction; PCD-CT140keV, virtual monoenergetic reconstruction at 140 keV; PCD-CTiMAR, dedicated iterative metal artifact reduction; and PCD-CT140keV+iMAR, the combination of iMAR with 140-keV virtual monoenergetic reconstruction [89].

Metal hardware produces complex CT artifacts through beam hardening, photon starvation, scatter, and corruption of projection data. High-keV virtual monoenergetic imaging and dedicated metal artifact reduction (MAR) algorithms address partly different components of this problem and may therefore provide complementary benefits. High-keV VMI reduces the attenuation differences responsible for beam-hardening and streak artifacts by synthesizing images at higher photon energies, whereas iterative MAR algorithms identify projections affected by metal and apply correction or interpolation strategies to reduce the resulting hypoattenuating and hyperattenuating streaks (Figure 12).

In photon-counting CT, combining these approaches has shown particular benefit for dense orthopedic and dental hardware. Experimental PCCT data demonstrated the greatest artifact reduction when iterative MAR was combined with approximately 110-keV VMI, while clinical studies of total hip arthroplasty have similarly identified approximately 110 keV as an effective compromise between artifact suppression and preservation of surrounding anatomy. In maxillofacial imaging, studies involving dental implants have shown that iterative MAR provides greater artifact suppression than high-keV VMI alone, while combining MAR with VMI at approximately 110 keV or higher can further improve visualization of tissues adjacent to metallic dental restorations and implants [90,91,92,93].

The optimal energy level is nevertheless task- and implant-dependent. Increasing the monoenergetic level does not produce progressively better images indefinitely; excessively high-keV reconstruction can reduce iodine and soft-tissue contrast and may introduce overcorrection or obscure diagnostically relevant structures. Consequently, high-keV VMI and MAR should be considered complementary reconstructions rather than automatic replacements for conventional images, and the optimal combination should be selected according to implant composition, size, anatomical region, and diagnostic target.

#### 8.3.2. Spectral Iodine Imaging: Perfusion-like Images, Maps, and Quantification

In contrast-enhanced spectral computed tomography (CT)—including photon-counting CT (PCCT)—material decomposition serves as the foundational physical and mathematical process that enables advanced iodine imaging. Because iodine possesses a characteristic, energy-dependent attenuation profile, spectral decomposition algorithms can accurately isolate its signal from background materials such as water-like soft tissue, calcium, hemorrhage, and metal. Perfusion-like iodine images, iodine maps, and iodine quantification all fall directly under the umbrella of material decomposition; they do not represent different acquisition techniques, but rather distinct tiers of data processing, visual display, and numerical analysis derived from the same decomposed spectral dataset. Understanding the operational boundaries between these three applications is critical to avoiding diagnostic misinterpretation.

Perfusion-like iodine images serve as visual, qualitative surrogates for tissue blood supply or contrast distribution. Typically displayed as color overlays on standard grayscale anatomical images, they estimate the spatial distribution of iodine within tissues at the exact moment of acquisition. Regions with reduced contrast delivery appear as low-signal or color-deficient areas, making them highly effective diagnostic screening tools.

Clinically, these images provide critical functional information that complements traditional anatomical findings. For instance, they can highlight wedge-shaped peripheral defects in pulmonary embolism, demonstrate reduced mural enhancement in bowel ischemia, evaluate renal perfusion, and map heterogeneous vascularity or necrosis in tumors [94,95].

However, a key limitation of perfusion-like iodine images is the potential for clinical overinterpretation. Because these images are typically derived from a single contrast-enhanced phase rather than time-resolved imaging, they are not true physiological perfusion maps. They do not provide dynamic kinetic parameters such as absolute blood flow, mean transit time, or vascular permeability. A reduced iodine signal may accurately reflect ischemia or vascular obstruction, but it can just as easily be an artifact of poor bolus timing, variation in cardiac output, motion, beam hardening, or partial volume effects.

Whereas perfusion-like images focus on the relative visual representation of blood supply, iodine maps are material-specific reconstructions that comprehensively chart the spatial distribution and density of iodine across the entire scanned volume. By separating the iodine signal from surrounding anatomical structures, iodine maps allow clinicians to definitively distinguish true contrast enhancement from high-attenuation background materials like intrinsic hemorrhage or calcification.

Iodine maps are invaluable problem-solving tools across multiple anatomical regions. In oncology, they support lesion characterization, tumor enhancement assessment, and the detection of residual viable tumor following therapy. In abdominal imaging, they may be used to evaluate liver lesions, renal masses, adrenal nodules, pancreatic tumors, and inflamed or ischemic bowel walls [96,97,98,99,100,101,102]. In chest imaging, they map the structural footprint of perfusion defects downstream of a pulmonary embolism.

**Figure 13:**
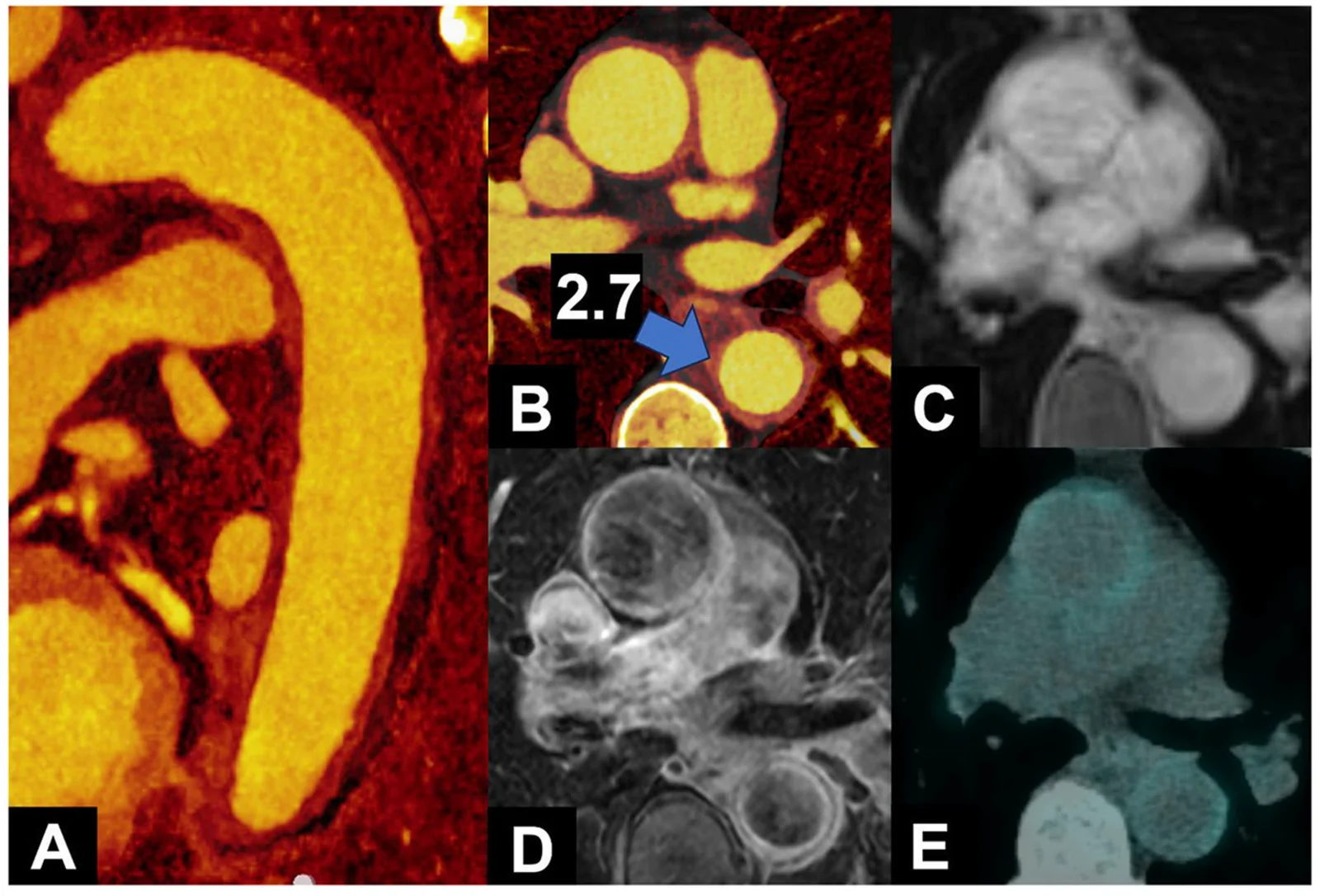
PCCT evaluation of the thoracic aorta in a 69-year-old woman during the venous phase. (**A**,**B**) Color-coded iodine maps show circumferential aortic wall thickening with iodine uptake of 2.7 mg I/mL. (**C**,**D**) T1-weighted late-enhancement and dark-blood MRI confirm the wall thickening and increased gadolinium enhancement. (**E**) Subsequent 18F-FDG PET/CT demonstrates increased tracer uptake, supporting the diagnosis of large-vessel vasculitis [103].

Iodine quantification represents the highest tier of spectral analysis, transitioning from the spatial visualization of iodine maps to absolute, objective numerical metrics. Depending on the scanner architecture and reconstruction algorithms, the localized concentration of iodine within a specific region of interest (ROI) can be extracted and measured, typically expressed in milligrams per milliliter (mg/mL) or related units (Figure 13).

By offering detector-level spectral data and significantly reducing electronic noise, PCCT has notably advanced the precision of these measurements. Clinically, this numerical analysis provides an objective framework to grade tissue ischemia, track longitudinal tumor responses to anti-angiogenic therapies, and differentiate benign from malignant lesions based on exact material density thresholds [104].

Despite the distinct clinical roles of these three modalities, they share common technical vulnerabilities. Crucially, single-phase iodine maps and their corresponding quantitative values reflect a frozen snapshot of iodine distribution at one specific time point, meaning they are highly dependent on contrast phase and injection protocols. Furthermore, quantitative accuracy relies heavily on proper detector calibration, spectral separation, reconstruction algorithms, and the selected material basis.

Apparent iodine defects or inaccurate quantitative values can be introduced by patient motion, respiratory misregistration, beam hardening, dense contrast artifacts, and partial volume averaging. Furthermore, quantitative iodine values cannot be assumed to be interchangeable across different vendors, scanner designs, acquisition protocols, or software thresholds. Consequently, to ensure diagnostic accuracy, perfusion-like images, iodine maps, and quantitative metrics must always be systematically correlated with baseline anatomical images, vascular findings, and the broader clinical context.

#### 8.3.3. Virtual Non-Contrast Images

Virtual non-contrast (VNC) images are spectral CT reconstructions generated from contrast-enhanced datasets after computational iodine subtraction. Their purpose is to approximate the appearance of a true non-contrast CT image without acquiring a separate unenhanced phase. In PCCT, this is possible because energy-resolved data allow iodine to be separated from background tissues using material decomposition algorithms.

VNC images are generated by identifying the iodine component within the contrast-enhanced acquisition and subtracting it from the dataset. The remaining image is intended to represent the non-iodine background attenuation of tissues. Because iodine attenuates X-rays differently from water-like soft tissue, calcium, fat, and other materials, the reconstruction algorithm can estimate and remove the iodine fraction within each voxel. However, this process is model-based and depends on detector calibration, spectral separation, contrast concentration, image noise, patient size, motion, material basis selection, and reconstruction algorithm.

VNC images resemble unenhanced CT images. Contrast-filled vessels, enhancing renal cortex, liver parenchyma, bowel wall, and enhancing organs should appear closer to baseline attenuation after iodine removal. However, VNC images are not identical to true non-contrast CT. Residual iodine may persist in regions of high iodine concentration, while calcifications, stones, dense hemorrhage, or small hyperattenuating lesions may be altered by the decomposition process [105,106,107]. VNC should therefore be regarded as a simulated non-contrast reconstruction rather than a perfect substitute for a separately acquired unenhanced scan. In this regard, it is worth mentioning PureCalcium, which is a specialized commercial post-processing algorithm (developed by Siemens Healthineers) designed specifically for PCCT to generate Virtual Non-Iodine (VNI) reconstructions. Conventional VNC algorithms struggle to differentiate between iodine and calcium often inadvertently erasing calcified plaques. By precisely isolating the distinct spectral signatures of each element VNI PureCalcium digitally subtracts the iodinated contrast medium while meticulously preserving the attenuation, volume, and geometry of calcifications. In a clinical and educational context, this technological synergy is a major milestone: it allows for accurate coronary artery calcium scoring and plaque burden assessment directly from a single, contrast-enhanced angiographic scan, potentially eliminating the need for a separate true non-contrast acquisition and significantly lowering the cumulative radiation dose for the patient [108].

In renal imaging, VNC may help distinguish true enhancement from intrinsic hyperattenuation in renal cysts or masses [109]. In adrenal imaging, it may support attenuation assessment, although conventional non-contrast HU thresholds should not be transferred automatically without validation [98,110]. More recent evidence suggests that In adrenal nodule characterization, Liver Virtual Noncontrast (LiverVNC) algorithms at venous phase photon-counting CT (PCCT) outperform air–soft tissue–iodine-based Virtual Unenhanced (VUE) algorithms [111].

In liver and oncologic imaging, VNC may reduce radiation exposure by avoiding a separate unenhanced phase in selected multiphasic protocols [96,112,113].

The most important pitfall is assuming that VNC is equivalent to true non-contrast CT. Incomplete iodine subtraction may leave residual hyperattenuation and falsely suggest hemorrhage, calcification, or intrinsic lesion density [114]. Conversely, structures that should remain hyperattenuating may be partially suppressed or misrepresented. This is particularly relevant in renal colic, urinary stone detection, intracranial hemorrhage evaluation, adrenal lesion characterization, and small hyperattenuating lesions. VNC should therefore be validated for each clinical task before it replaces true non-contrast imaging.

#### 8.3.4. Calcium Subtraction Techniques

In spectral CT calcium-subtracted (vascular) and virtual non-calcium (VNCa/musculoskeletal) images represent distinct reconstruction pipelines that apply spectral material decomposition to solve unique clinical problems. While both techniques exploit the energy-resolved attenuation profile of calcium hydroxyapatite, they differ completely in their algorithmic targets, neighboring tissues, and visual outputs. Calcium-subtracted imaging on PCCT isolates and erases focal, high-density calcified vascular plaques to reduce blooming artifacts and reveal the true internal lumen diameter in coronary or carotid CT angiography, differentiating calcium directly from adjacent iodine [115,116]. Shifting inward to the skeleton, virtual non-calcium imaging as demonstrated using Dual Energy CT (DECT) targets diffuse, low-density cancellous bone to separate calcium from marrow fat and water; instead of deleting a structure, it peels back the mineralized mesh to unmask the underlying soft-tissue attenuation, producing grayscale or color-coded maps that reveal marrow edema, occult fractures, or tumor infiltrates [117,118].

Bone marrow edema, which may be inconspicuous on conventional CT because of the superimposed attenuation of trabecular bone, can consequently become more readily detectable on calcium-suppressed reconstructions. Potential applications include evaluation of acute and occult fractures, bone contusions, vertebral compression fractures, inflammatory arthropathies, and marrow infiltration. The majority of the evidence supporting VNCa for bone marrow edema detection has historically been derived from DECT, where calcium-suppressed reconstructions have demonstrated diagnostic utility across several anatomical regions, often using MRI as the reference standard. These data have established the underlying principle that spectral separation of mineralized bone from marrow can reveal abnormalities that are poorly conspicuous on conventional CT. However, diagnostic thresholds and performance established using DECT should not be assumed to transfer directly to photon-counting CT because detector architecture, spectral sampling, material-decomposition algorithms, reconstruction parameters, and quantitative output differ between platforms.

Emerging PCCT-specific evidence nevertheless suggests that marrow characterization may become an important musculoskeletal application [47]. The improved spatial and low-contrast resolution of PCCT, together with energy-resolved material decomposition, may improve characterization of the interface between fatty marrow, cellular marrow, and mineralized trabecular bone and increase the conspicuity of bone marrow edema and contusions. This capability may be particularly valuable in trauma patients when MRI is unavailable, contraindicated, or impractical [119].

**Figure 14:**
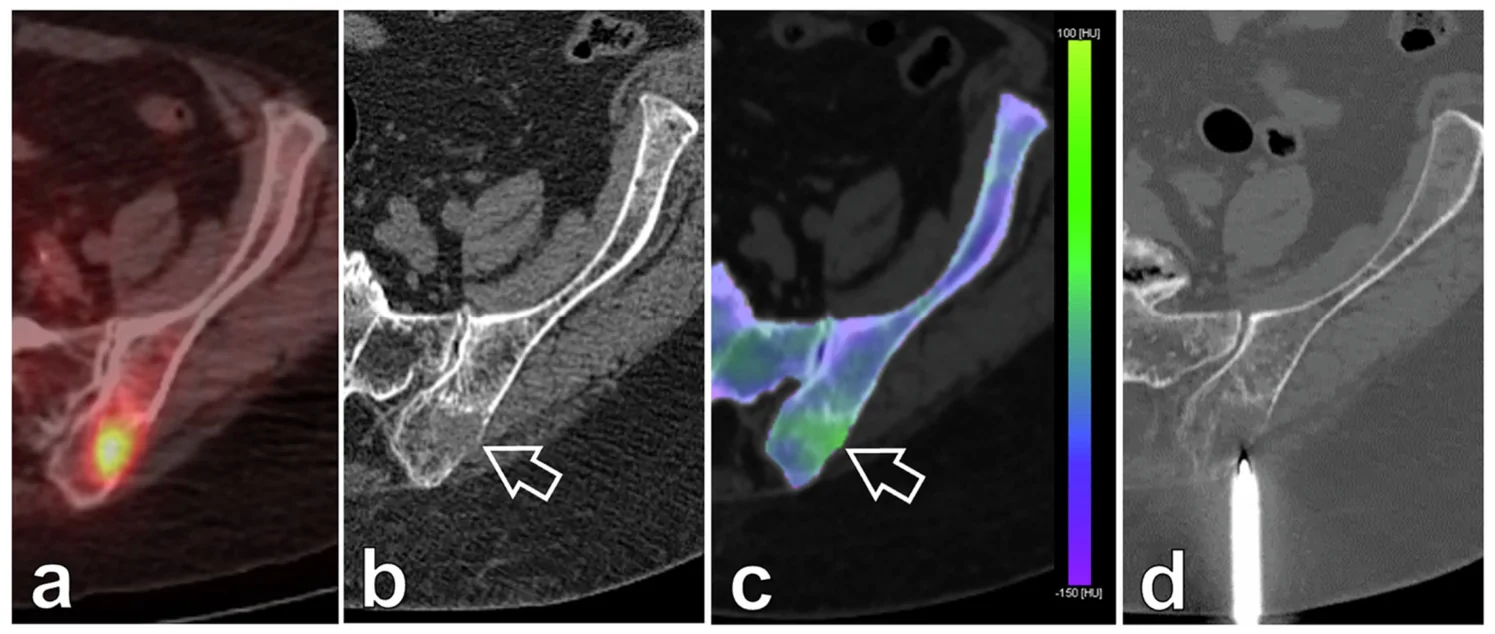
An 89-year-old woman with stage IV breast cancer underwent PCCT-guided biopsy of a suspicious left iliac lesion identified on follow-up PET/CT (**a**). (**b**) Axial 70-keV monoenergetic bone reconstruction demonstrates an ill-defined heterogeneous lytic lesion in the posterior left iliac blade (arrow). (**c**) Real-time bone marrow oedema (BME) mapping shows abnormal increased values within the lesion, displayed in yellow/green, compared with normal marrow in blue/violet. (**d**) BME mapping enabled precise intraprocedural targeting of the abnormal region for biopsy. Histopathology confirmed metastatic breast cancer. BME, bone marrow oedema; PCCT, photon-counting computed tomography [119].

More recently, PCCT-derived bone marrow edema maps have also been investigated for interventional applications. In an exploratory clinical study of PCCT-guided bone biopsy, real-time BME mapping was used to improve visualization of heterogeneous osseous lesions and assist targeting of clinically active regions for tissue sampling [119]. The approach was feasible and was associated with improved lesion visualization, procedural targeting, and operator confidence, suggesting that spectral marrow information may have value not only for diagnosis but also for image-guided intervention (Figure 14). However, these findings remain preliminary and require validation in larger prospective cohorts.

Consequently, PCCT-based VNCa and related marrow maps should currently be regarded as promising complementary reconstructions rather than established replacements for MRI. Prospective studies comparing PCCT directly with MRI are needed to define sensitivity and specificity for bone marrow edema, optimal calcium-suppression parameters, quantitative thresholds, inter-reader reproducibility, and performance across traumatic, inflammatory, neoplastic, and interventional applications.

### 8.4. Quantitative Reconstructions

#### 8.4.1. Effective Atomic Number Maps

Effective atomic number (Zeff) maps are quantitative spectral reconstructions that estimate the atomic-number-dependent attenuation characteristics of materials or tissue mixtures rather than assigning each voxel a true elemental atomic number. Zeff can be calculated from attenuation measurements acquired at different photon energies and may be evaluated together with electron density to support material classification and multi-material separation [44,120,121].

Phantom testing using a prototype deep-silicon photon-counting CT system showed that Zeff measurements were generally more accurate and more consistent across different water-equivalent object sizes than measurements obtained with dual-energy energy-integrating detector CT. However, the magnitude of error varied according to the tested material, indicating that Zeff accuracy remains dependent on material composition, object size, detector architecture, and calibration method [44].

Physics-based modeling may further improve Zeff estimation. Dong et al. proposed a model for calculating effective atomic number and effective electron density from photon-counting CT data and reported relative standard deviations below 1% in simulations. Their combined Zeff representation also allowed five simulated materials to be separated, suggesting potential value for simultaneous multi-material identification [120].

Similarly, Sakurai et al. derived Zeff and electron density from measured X-ray attenuation coefficient spectra using experimental calibration with standard materials. Their method achieved approximately 1.1% accuracy for Zeff when the calibration materials had atomic numbers similar to those of the evaluated samples, emphasizing the importance of calibration-material selection [121].

Early clinical evidence also supports the feasibility of Zeff -based coronary plaque analysis. In 64 plaques from 10 patients, Asahara et al. found strong positive correlations between a Zeff -based plaque score and both the Agatston score and mean coronary artery calcium score. Unlike conventional calcium scoring, which includes only voxels exceeding 130 HU, the Zeff score incorporated all voxels within the plaque region; conventional scores excluded approximately 39% of the plaque area in that study. The authors therefore proposed that continuous Zeff analysis may characterize both lower- and higher-density components across the entire plaque volume [122].

Nevertheless, the available evidence remains preliminary. Several studies were phantom-based, simulation-based, or performed using prototype or laboratory systems, and Zeff values should not yet be assumed to be interchangeable across detector designs, reconstruction algorithms, calibration procedures, energy-bin configurations, object sizes, or clinical applications [44,120,121,122].

#### 8.4.2. Electron Density Maps

Electron density maps estimate the number of electrons per unit volume within tissues. Electron density is relevant because Compton scattering, which contributes substantially to X-ray attenuation in soft tissues, is closely related to electron density, while photoelectric absorption is more strongly influenced by atomic composition.

In spectral CT, attenuation is measured at multiple energy levels. Reconstruction algorithms then model the measured energy-dependent attenuation as a combination of different physical contributions, commonly represented by a Compton-related component and a photoelectric-related component, or by selected basis materials. From this model, the scanner can estimate electron density and effective atomic number for each voxel. These values are therefore derived quantities rather than direct detector measurements [123].

Experimental work supports the quantitative feasibility of this approach in photon-counting CT. In a phantom study using eight tissue-equivalent materials, Son et al. applied stoichiometric calibration to photon-counting CT data and reported mean relative electron density errors of 1.68% using energy-bin images, compared with 4.58% using full-spectrum images. Errors for both effective atomic number and relative electron density in energy-bin mode remained within 4%, suggesting that energy-resolved photon-counting data may improve quantitative electron-density estimation when appropriate calibration is used [124]. However, the study was performed using a dedicated experimental system and identified ring artifacts and detector-pixel nonuniformity as technical limitations.

Although electron density maps are not commonly used for routine diagnostic reporting, they are relevant to radiotherapy planning, dose calculation, and quantitative imaging research. In radiotherapy planning, they provide direct physical information for photon-dose calculation. In a proof-of-concept study of 29 contrast-enhanced abdominal PCCT examinations with simulated pancreatic treatment plans, dose calculations based on 70-keV virtual monoenergetic images and electron-density (Rho) images showed close agreement, with most dose-volume histogram differences within 1%, a maximum difference of −1.60%, and gamma pass rates above 98% in most cases [125,126]. Although 70-keV VMI required a dedicated Hounsfield-unit-to-relative-electron-density calibration curve, Rho images permitted direct conversion and suppressed iodine-related contrast enhancement, potentially reducing uncertainty in contrast-enhanced tissues. These findings suggest that PCCT-derived electron-density maps may support accurate radiotherapy dose calculation while retaining the anatomical and spectral advantages of PCCT, although further validation is required across larger cohorts, anatomical regions, scanner platforms, and treatment-planning settings.

#### 8.4.3. K-Edge Imaging

K-edge imaging is a spectral CT technique that identifies specific high-atomic-number materials from the abrupt increase in their X-ray attenuation when photon energy exceeds the K-shell binding energy. Unlike conventional material decomposition, which separates materials according to broader differences in their energy-dependent attenuation, K-edge imaging aims to exploit this element-specific discontinuity. It is therefore particularly attractive for distinguishing multiple contrast agents within the same acquisition and for developing molecular, functional, and theranostic imaging applications [127].

Recent phantom work has demonstrated that K-edge imaging can be performed on clinical or near-clinical photon-counting CT systems. Rybertt et al. used a clinical dual-source PCCT scanner with four energy thresholds to simultaneously decompose iodine and gadolinium in pure and mixed solutions. Quantification was feasible across concentrations of 1–10 mg/mL and doses of 1–8 mGy, although accuracy was influenced by radiation dose, contrast concentration, and whether the agents were present alone or in mixtures. Higher dose and concentration improved quantitative performance, while mixed-agent solutions showed lower contrast-to-noise performance than pure solutions [128]. Similarly, Coulibaly et al. demonstrated separation of iodine and gadolinium using three-material decomposition in a spectral phantom, including at concentrations as low as 0.5 mg/mL. However, both agents were underestimated, with greater gadolinium underestimation in mixed solutions, indicating that material separation does not yet guarantee fully accurate quantification [129].

The selection and positioning of energy thresholds are central to K-edge performance. Energy bins must adequately sample attenuation below and above the relevant K-edge while preserving sufficient photon statistics. Optimized bin-selection methods that account for the continuous X-ray spectrum and imperfect detector energy resolution may strengthen the extracted K-edge signal, particularly at low contrast-agent concentrations, and could potentially permit reduced contrast doses [130]. Nevertheless, some K-edge applications remain technically challenging. Iodine is especially difficult because its K-edge lies at a relatively low energy, where photon attenuation is high and three-material decomposition becomes sensitive to noise and calibration errors. Simulation work has therefore explored deep-learning approaches that combine multiple two-basis decompositions to generate iodine and tissue basis images, although such findings remain preclinical and require validation under realistic noise conditions [131].

High-atomic-number contrast agents other than iodine may be better suited to K-edge imaging because their K-edges can lie within more favorable portions of the diagnostic spectrum. Proposed agents include gadolinium, tungsten, tantalum, bismuth, gold, and other lanthanide-based materials. Potential applications include dual-contrast imaging, cardiovascular plaque characterization, stent visualization, targeted molecular imaging, and theranostic imaging [127]. However, most of these agents remain experimental, and clinical translation depends not only on spectral detectability but also on biocompatibility, pharmacokinetics, toxicity, regulatory approval, and the availability of robust decomposition algorithms.

**Figure 15:**
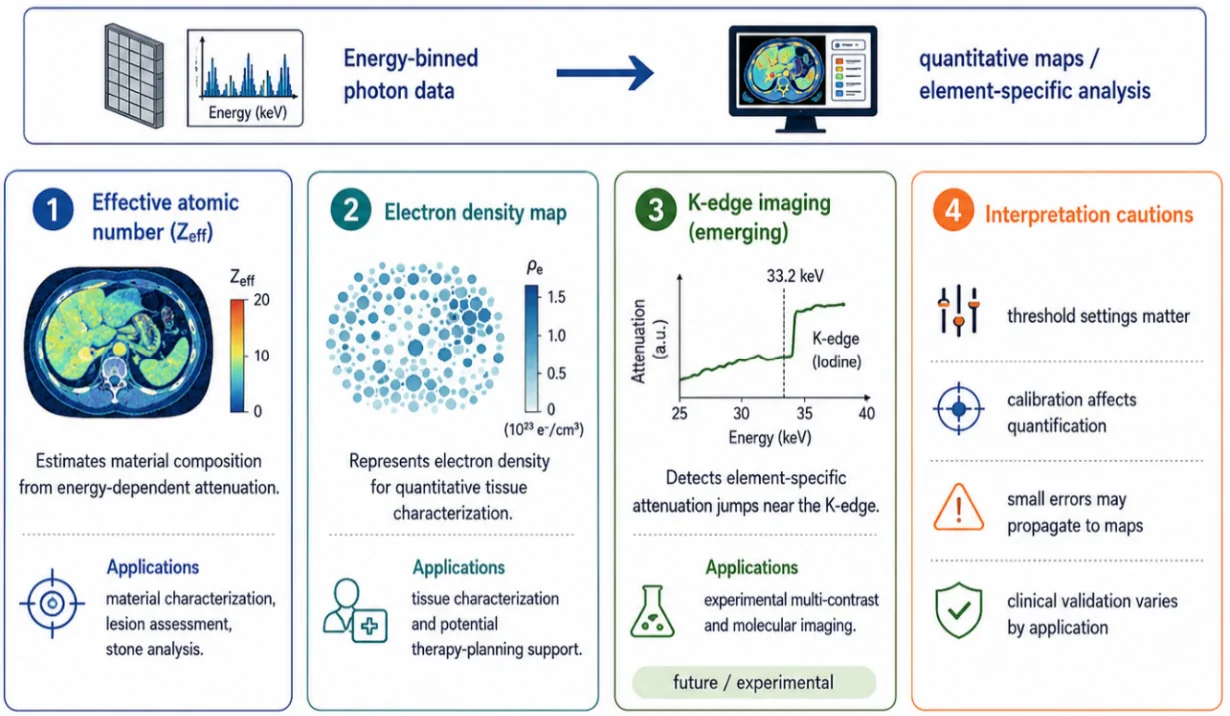
Quantitative photon-counting CT reconstructions. Energy-binned data can generate effective atomic number and electron-density maps for quantitative tissue characterization, while emerging K-edge imaging identifies element-specific attenuation signatures; accuracy depends on threshold selection, calibration, and application-specific validation.

K-edge contrast agents may also introduce important artifacts. In a phantom comparison, Pourmorteza et al. found greater beam-hardening artifacts on PCD-CT than on EID-CT at tube voltages of 120 kVp and above, with particularly complex behavior for gadolinium- and bismuth-based agents. Although PCCT-derived virtual monoenergetic imaging eliminated iodine-related beam-hardening artifacts in that experiment, special correction strategies were still required for non-iodine K-edge agents [132]. Therefore, K-edge maps should currently be interpreted as experimental material-specific reconstructions whose accuracy depends on energy-threshold design, detector energy resolution, dose, contrast concentration, material mixtures, calibration, beam-hardening correction, and decomposition method (Figure 15).

### 8.5. Clinical–Experimental Roadmap of PCCT Reconstructions

PCCT applications span a continuum from reconstructions already integrated into routine clinical workflows to techniques that remain emerging or predominantly investigational. Conventional polyenergetic images, virtual monoenergetic imaging, iodine maps, and selected virtual non-contrast or calcium-subtraction applications are already used clinically on currently available PCCT systems. In contrast, advanced multi-material decomposition, quantitative Zeff and electron-density applications beyond selected validated tasks, virtual non-calcium marrow imaging in PCCT, and especially K-edge imaging remain less standardized and require further technical and clinical validation. Table 4 summarizes this practical roadmap.

## 9. Practical Implementation and Interpretation Considerations

The clinical value of PCCT depends not only on detector performance but also on selecting acquisition and reconstruction settings that match the diagnostic question. Protocols should therefore define the acquisition mode, tube voltage, dose target, primary reconstruction energy, section thickness, kernel, iterative reconstruction strength, matrix size, and spectral outputs to be archived. These choices should be established before scanning rather than left entirely to retrospective post-processing.

### 9.1. Protocol and Reconstruction Selection

Tube voltage and intended virtual monoenergetic image energy should be planned together. Higher tube voltage may improve photon penetration and spectral separation, particularly in larger patients or photon-starved regions, while lower-keV reconstruction can increase iodine conspicuity. The selected keV level should therefore be considered part of the acquisition strategy because dose modulation and image-quality targets may be optimized for the intended primary reconstruction. Interpreting the examination mainly at a different energy may produce an unintended balance between contrast, noise, and dose. Conventional HU thresholds should also not be transferred directly to monoenergetic images without validation because measured attenuation varies with the selected keV level.

Not every acquisition mode provides every spectral output. Depending on the scanner, protocol, and archived dataset, an examination may permit VMI but not necessarily iodine maps, virtual non-contrast images, calcium subtraction, or other material-specific reconstructions. The required outputs should therefore be identified before scanning. For example, pulmonary embolism imaging may require low-keV VMI and iodine maps, renal or adrenal characterization may benefit from VNC images, musculoskeletal trauma may require ultra-high-resolution or virtual non-calcium images, and vascular imaging in the presence of dense calcification may benefit from calcium-subtracted reconstructions.

Protocol adaptation remains necessary in larger and pediatric patients. In larger patients, adequate photon penetration may require higher tube voltage or dose targets, with low-keV VMI subsequently used to recover iodine contrast; excessively low-keV images may nevertheless become noisy when photon statistics are insufficient. In pediatric and repeatedly imaged patients, the dose efficiency and spatial resolution of PCCT may support reductions in radiation or contrast-media burden, but parameters should be adjusted to patient size and the specific diagnostic task [76,133,134,135]. The objective is not to generate the maximum number of reconstructions, but to obtain the required diagnostic information with the lowest reasonable radiation dose, contrast dose, and image burden.

Recent clinical studies illustrate the magnitude of these potential reductions, although the achievable benefit varies substantially according to anatomical region, patient size, acquisition settings, reconstruction technique, and diagnostic task. Compared with conventional energy-integrating detector CT, clinical PCCT protocols have reported radiation-dose reductions of approximately 24–66% while maintaining diagnostic image quality, with greater reductions demonstrated in selected low-dose experimental or task-specific settings. Similarly, reductions in iodinated contrast administration of approximately 20–60% have been reported in abdominal, thoracic, and angiographic applications, particularly when low-keV virtual monoenergetic reconstructions are used to preserve or increase iodine conspicuity [75,136,137,138,139,140]. These values should therefore be regarded as representative protocol-specific results rather than universal dose-saving capabilities of PCCT.

### 9.2. Spatial Resolution and Reconstruction Parameters

Ultra-high-resolution imaging should be prescribed selectively when fine anatomical detail is central to the clinical question, such as in temporal bone, lung, coronary artery, stent, small-vessel, and musculoskeletal imaging. The thinnest sections and sharpest kernels improve edge definition but increase noise, image volume, reconstruction time, and storage requirements. Ultra-high-resolution images should therefore complement rather than replace routine soft-tissue reconstructions.

Kernel and iterative reconstruction settings should also be task-specific. Sharp kernels preserve small structures but increase image noise, whereas stronger iterative reconstruction reduces noise but may smooth fine detail or alter texture. Matrix sizes of 768 × 768 or 1024 × 1024 may improve in-plane sampling when the field of view and detector resolution support it, but institutions should confirm compatibility with PACS and third-party software used for cardiovascular, orthopedic, quantitative, or radiotherapy applications [141,142]. When both maximum spatial resolution and spectral analysis are required, separate anatomical and spectral series may be necessary.

### 9.3. PACS, Archiving, and Workflow

PCCT can generate many more series than conventional CT, but routinely sending every available reconstruction to PACS may increase interpretation burden and storage demands without improving diagnosis. A practical protocol should define one primary anatomical series and a limited number of task-specific supplementary reconstructions [112]. Examples include a standard or 60–70-keV abdominal series with iodine maps when enhancement assessment is required, low-keV images and iodine maps for pulmonary angiography, ultra-high-resolution bone images for musculoskeletal examinations, and high-keV or calcium-subtracted images for vascular studies affected by dense material.

The underlying spectral dataset should be archived when retrospective generation of additional reconstructions is clinically desirable. Saving only conventional reconstructed images may eliminate the opportunity for later spectral post-processing. Clear policies are therefore needed for data retention, series naming, display order, PACS transmission, and compatibility with post-processing platforms.

### 9.4. Interpretation Workflow

A systematic review sequence helps prevent overreliance on any single reconstruction. The radiologist should first evaluate the conventional polyenergetic or primary monoenergetic series for anatomy, contrast phase, overall image quality, and artifacts. Ultra-high-resolution images can then be reviewed for fine structural detail. Low-keV images are used when greater iodine conspicuity is needed, whereas high-keV images are useful for reducing beam-hardening, dense-contrast, or metal-related artifacts. Iodine maps, VNC images, virtual non-calcium images, calcium-subtracted images, and quantitative maps should be selected according to the diagnostic question and interpreted alongside the anatomical source images.

Window settings may need adjustment, particularly at low keV, where vessels and enhancing tissues can appear substantially brighter than on conventional CT. Quantitative measurements and established HU thresholds should be applied only to the reconstruction type and energy level for which they have been validated. Material-specific maps should be treated as complementary reconstructions because motion, noise, partial volume, subtraction errors, contrast timing, and calibration can produce false-positive or false-negative findings (Table 5).

## 10. Conclusions

Photon-counting CT extends CT beyond conventional attenuation imaging by combining high-resolution anatomical acquisition with energy-resolved and quantitative reconstruction. Its advantages arise from direct photon detection, smaller detector pixels, electronic-noise rejection, and the preservation of energy-dependent attenuation information. These detector-level features support ultra-high-resolution imaging, virtual monoenergetic reconstruction, material-specific maps, and quantitative outputs such as effective atomic number and electron density.

The clinical value of PCCT nevertheless depends on selecting and interpreting these outputs appropriately. Conventional or primary monoenergetic images remain the anatomical reference, whereas low- and high-keV images, iodine maps, virtual non-contrast images, subtraction techniques, and quantitative maps should be used to answer specific diagnostic questions. None is universally interchangeable with conventional CT, and each is influenced by acquisition settings, photon statistics, detector nonidealities, calibration, reconstruction algorithms, motion, partial volume, and material-decomposition errors; this inter-platform variability remains an important challenge for multicenter studies and highlights the need for harmonized acquisition, reconstruction, and calibration standards.

PCCT should therefore be implemented as a task-specific imaging platform rather than as a means of generating every available reconstruction. Protocols must define the required spectral outputs, primary interpretation series, reconstruction parameters, and data-archiving strategy before acquisition. Wider global adoption will also depend on continued engineering advances, including faster high-count-rate ASIC electronics with improved pulse processing, more robust correction of detector nonidealities, and scalable, cost-effective production of high-quality semiconductor detector materials. Reducing system complexity and manufacturing cost while maintaining spectral accuracy and detector stability will be important for extending PCCT beyond high-resource centers [63]. Further work is needed to standardize protocols and terminology, validate quantitative biomarkers, establish cross-vendor comparability, and determine when PCCT reconstructions provide clinically meaningful benefits over established CT methods. With these safeguards, PCCT has the potential to improve structural visualization, material characterization, quantitative imaging, and dose-efficient clinical practice.

## Figures and Tables

**Table 1:** Energy-integrating CT versus photon-counting CT.

Feature	Energy-Integrating CT	Photon-Counting CT	Clinical Consequence
Detector mechanism	Indirect conversion	Direct conversion	Less light spread
Signal	Total energy	Individual photons	Energy information preserved
Electronic noise	Integrated	Threshold-rejected	Better low-dose imaging
Spectral imaging	Requires dual-source/dual-kVp/other methods	Intrinsic energy binning	Material decomposition
Spatial resolution	Limited by scintillator/light spread	Smaller pixels possible	Better small structure imaging
Dose efficiency	Septa reduce active area	Less need for septa	Improved dose use
Quantification	Limited	Material-specific maps	Iodine/Zeff/electron density

**Table 2:** Representative commercial and experimental photon-counting CT systems.

System/Manufacturer	Status	Detector Material/Design	Energy Bins/Thresholds	Threshold Configuration	Representative Design Characteristics
NAEOTOM Alpha/Siemens Healthineers	Commercial clinical dual-source PCCT	CdTe direct-conversion photon-counting detectors; dual-source architecture [32,34].	Energy-threshold configuration is acquisition-mode dependent. Published clinical studies report two-threshold operation, while four-threshold research configurations have also been investigated [35,36].	A documented 120-kVp clinical configuration uses thresholds at 20 and 65 keV [35]. A four-threshold research mode has been used for multi-material/K-edge studies [36].	Platform-specific UHR capability and high spatial resolution are described in the manuscript [37,38,39,40,41].
Photonova Spectra/GE HealthCare	Commercial PCCT platform; FDA-cleared in 2026	Edge-on silicon (‘Deep Silicon’) photon-counting detector architecture; silicon-based PCCT principles are discussed in the manuscript [31,32,42,43,44].	8 energy bins [45].	GE publicly specifies 8-bin spectral imaging, but individual threshold energies are not publicly disclosed in the manufacturer material reviewed; no threshold positions are inferred [45].	Deep Silicon edge-on architecture; manufacturer-reported 8-bin spectral imaging and platform-specific detector coverage/features [45].
Spectral photon-counting CT clinical prototype/Philips	Clinical research prototype	CZT direct-conversion detector architecture; CZT detector properties and photon-counting principles are discussed in the manuscript [32,34,42,46,47].	5 energy bins/5 thresholds in the published whole-body clinical prototype [48].	Published prototype configuration: 30, 51, 62, 72, and 81 keV [48]. Earlier experimental configurations may use different task-specific thresholds.	Whole-body clinical research prototype with multi-energy acquisition and material-decomposition capability consistent with the spectral principles discussed in the manuscript [49,50,51,52,53].
Photon-counting CT prototype/Canon Medical Systems–Redlen	Experimental/under development	CZT direct-conversion detector architecture; CZT detector physics are discussed in the manuscript [32,34,42,46,47].	Configurable energy bins; a fixed public bin count was not identified in the manufacturer material reviewed [54].	Canon states that energy-bin thresholds are configurable and can be selected for specific spectral/K-edge tasks; exact routine threshold positions are not publicly specified [54].	High-Z CZT direct conversion with configurable spectral thresholds; manufacturer describes ongoing PCCT development rather than a standardized fixed clinical configuration [54].

**Table 3:** Technical challenges in PCCT.

Challenge	Mechanism	Image Effect	Clinical Implication	Mitigation
Charge sharing	Charge spreads to neighboring pixels	Spatial/spectral distortion	Affects small structures and material maps	Charge summing/correction
Pulse pile-up	Multiple photons counted as one	Count loss, energy error	High-flux artifacts	Fast electronics/correction
K-escape	Fluorescent photon escapes detector site	Energy misclassification	Quantification error	Calibration/correction
Count-rate limits	Detector overwhelmed	Nonlinear response	Large patients/cardiac CT	Flux management
Threshold drift	Energy thresholds unstable	Ring/spectral artifacts	Material map error	Calibration
Electronic noise	Low-amplitude noise	Low-dose degradation	Reduced in PCCT	Lower threshold rejection

**Table 4:** Practical roadmap of photon-counting CT applications: routine clinical use versus emerging and experimental domains.

Application/Reconstruction	Roadmap Category	Current Practical Role	Representative Applications	Principal Limitations/Evidence Gap
Conventional polyenergetic/primary anatomical images [49,50,51,66]	Routine clinical	Primary anatomical reference series derived from PCCT data.	Routine anatomical interpretation and comparison with prior CT examinations.	Does not fully display the spectral or material-specific information contained in the acquisition [49,50,51,66].
Virtual monoenergetic imaging (VMI) [49,50,51,53,69,70,71,72,73,74,75,76,77,81,82]	Routine clinical	Established spectral reconstruction selected according to the diagnostic task.	Low-keV: increased iodine conspicuity in CTA and contrast-enhanced abdominal/oncologic imaging [69,70,71,72,73,74,75,76,77]. High-keV: reduction in beam-hardening, dense-contrast and metal-related artifacts [74,81,82,90,91,92,93].	Optimal keV is task-, patient- and protocol-dependent; very low keV may increase noise, whereas high keV reduces iodine conspicuity [74,81,82].
Ultra-high-resolution (UHR) reconstruction [37,38,39,40,41,67,68]	Routine clinical on selected PCCT platforms/indications	Targeted high-spatial-resolution anatomical reconstruction.	Lung interstitium, temporal bone, coronary arteries/stents, small vessels, bone and urinary stones [37,38,67,68].	Very thin sections and sharp kernels increase noise and data burden; technical capabilities are platform-specific [37,38,67,68].
Iodine maps/iodine quantification [94,95,96,97,98,99,100,101,102,104]	Clinical; increasingly established but task- and platform-dependent	Material-specific visualization and quantitative assessment of iodine distribution.	Pulmonary perfusion-like defects, tumor enhancement/response, ischemia, and renal/hepatic lesion characterization [94,95,96,97,98,99,100,101,102,104].	Single-phase iodine distribution is not true dynamic perfusion; values depend on contrast timing, calibration, motion, partial volume and reconstruction implementation [94,95,104].
Virtual non-contrast (VNC) [96,98,105,106,107,109,110,111,112,113,114]	Clinical, selected/task-dependent	Computational iodine subtraction to approximate an unenhanced image from a contrast-enhanced dataset.	Selected renal, adrenal, liver and multiphasic abdominal/oncologic applications [96,98,109,110,111,112,113].	Not identical to true non-contrast CT; residual iodine or subtraction errors may alter calcification, hemorrhage, stones or lesion attenuation [105,106,107,114].
Calcium-subtracted vascular imaging [115,116]	Clinical/selected vascular applications	Spectral separation/removal of calcium to improve visualization of the contrast-enhanced vascular lumen.	Calcified coronary and selected vascular CTA applications [115,116].	Susceptible to motion and subtraction errors and to incomplete separation of iodine and calcium [115,116].
Virtual non-calcium (VNCa)/bone-marrow maps [47,117,118,119]	Emerging/investigational in PCCT	Calcium-suppressed marrow assessment.	Bone marrow edema, occult fractures and marrow abnormalities; exploratory PCCT-guided biopsy targeting [47,117,118,119].	Most established diagnostic evidence derives from DECT; PCCT-specific evidence remains limited and preliminary [47,117,118,119].
Effective atomic number (Zeff) maps [44,120,121,122]	Emerging quantitative/research	Quantitative characterization of atomic-number-dependent attenuation.	Material characterization and exploratory coronary plaque/tissue-composition assessment [44,120,121,122].	Evidence remains largely phantom-, simulation-, prototype- or small-cohort based; calibration dependence and limited cross-platform standardization remain important [44,120,121,122].
Electron-density (Rho) maps [123,124,125,126]	Specialized/emerging clinical and research	Quantitative estimation of electron density, with particular relevance to radiotherapy.	Radiotherapy dose calculation and quantitative imaging research [123,124,125,126].	Limited routine diagnostic use; broader validation across platforms and clinical settings is required [124,125,126].
Advanced multi-material decomposition [24,53,63,83,84]	Emerging/experimental	Separation or estimation of multiple material components from energy-resolved attenuation data.	Advanced material characterization and multi-material spectral analysis [24,53,63,83,84].	More complex than two-material decomposition and sensitive to noise, mixed voxels, calibration, material-basis selection and reconstruction method [53,63,83,84].
K-edge imaging [127,128,129,130,131,132]	Experimental	Element-specific spectral identification based on the abrupt attenuation change at a material’s K-edge.	Dual-/multi-contrast imaging and potential molecular, targeted and theranostic applications [127,128,129,130,131].	Accuracy depends on threshold placement, dose, concentration, detector energy resolution, calibration, material mixtures and beam-hardening correction; most non-iodine agents remain experimental [127,128,129,130,131,132].

**Table 5:** Summary of major photon-counting CT reconstructions and clinical applications.

Reconstruction	Main Clinical Applications	Principal Benefit	Key Limitations
Conventional polyenergetic images	Routine anatomical interpretation and comparison with prior CT examinations	Familiar CT appearance with potential gains in dose efficiency and spatial resolution	Does not fully display spectral or material-specific information
Ultra-high-resolution images	Lung interstitium, temporal bone, coronary arteries and stents, small vessels, bone, and urinary stones	Improved visualization of fine anatomical detail and small structures	Higher image noise, larger datasets, and greater reconstruction and storage burden
Low-keV virtual monoenergetic images	CT angiography, oncologic and abdominal lesion detection, and examinations with reduced or suboptimal iodine enhancement	Increased iodine conspicuity and may improve contrast-to-noise ratio	Noise and artifacts may increase at very low keV; conventional HU thresholds may not apply
High-keV virtual monoenergetic images	Metal hardware, dense contrast, calcification, skull base, shoulders, pelvis, and posterior fossa	Reduction in beam-hardening, dense-contrast, and metal-related artifacts	Reduced iodine conspicuity and lower sensitivity for subtle enhancement
Iodine maps and iodine quantification	Pulmonary perfusion defects, tumor enhancement and response, ischemia, and renal or hepatic lesion characterization	Material-specific visualization and scanner- and protocol-dependent quantitative assessment of iodine distribution	Strong dependence on contrast timing, calibration, motion, partial volume, and vendor-specific algorithms
Virtual non-contrast images	Renal and adrenal lesion assessment and selected multiphasic abdominal or oncologic protocols	May reduce the need for a separate unenhanced acquisition in selected applications	Not equivalent to true non-contrast CT; residual iodine or erroneous subtraction may alter calcification, hemorrhage, or stones
Calcium-subtracted vascular images	Coronary and selected vascular CT angiography applications in the presence of dense calcification	Reduced calcium blooming and improved evaluation of the contrast-enhanced lumen	Subtraction errors, motion, and incomplete separation of iodine and calcium
Virtual non-calcium images	Bone marrow edema, occult fractures, and marrow infiltration	Suppresses mineralized bone to reveal marrow abnormalities	Evidence is mainly derived from dual-energy CT; PCCT-specific validation remains limited
Effective atomic number maps	Material characterization and emerging plaque or tissue-composition assessment	Provides quantitative information related to atomic-number-dependent attenuation	Calibration dependent; limited standardization and cross-platform comparability
Electron-density maps	Radiotherapy planning, dose calculation, and quantitative research	Provides a quantitative physical parameter relevant to dose calculation	Limited routine clinical use and dependence on system-specific calibration
K-edge imaging	Experimental dual-contrast, molecular, and targeted imaging, with potential future theranostic applications	Potential element-specific identification of high-atomic-number contrast agents	Currently experimental; sensitive to threshold selection, dose, concentration, calibration, and beam hardening

## Data Availability

No new data were created or analyzed in this study. Data sharing is not applicable to this article.
